# Adaptive Fast Non-Singular Terminal Sliding Mode Path Following Control for an Underactuated Unmanned Surface Vehicle with Uncertainties and Unknown Disturbances

**DOI:** 10.3390/s21227454

**Published:** 2021-11-10

**Authors:** Yunsheng Fan, Bowen Liu, Guofeng Wang, Dongdong Mu

**Affiliations:** 1 School of Marine Electrical Engineering, Dalian Maritime University, Dalian 116026, China; bowenliu@dlmu.edu.cn (B.L.); dmuwgf@dlmu.edu.cn (G.W.); ddmu@dlmu.edu.cn (D.M.); 2 Key Laboratory of Technology and System for Intelligent Ships of Liaoning Province, Dalian 116026, China

**Keywords:** unmanned surface vehicle, path following, line-of-sight, sensor application, fast non-singular terminal sliding mode control

## Abstract

This paper focuses on an issue involving robust adaptive path following for the uncertain underactuated unmanned surface vehicle with time-varying large sideslips angle and actuator saturation. An improved line-of-sight guidance law based on a reduced-order extended state observer is proposed to address the large sideslip angle that occurs in practical navigation. Next, the finite-time disturbances observer is designed by considering the perturbations parameter of the model and the unknown disturbances of the external environment as the lumped disturbances. Then, an adaptive term is introduced into Fast Non-singular Terminal Sliding Mode Control to design the path following controllers. Finally, considering the saturation of actuator, an auxiliary dynamic system is introduced. By selecting the appropriate design parameters, all the signals of the whole path following a closed-loop system can be ultimately bounded. Real-time control of path following can be achieved by transferring data from shipborne sensors such as GPS, combined inertial guidance and anemoclinograph to the Fast Non-singular Terminal Sliding Mode controller. Two examples as comparisons were carried out to demonstrate the validity of the proposed control approach.

## 1. Introduction

The Unmanned Surface Vehicle (USV) has the advantages of small volume, multi-purpose, intelligence, etc. Whether in the military or civilian field, it has a great application prospect [1]. Whether the USV can accomplish specific tasks in a complex marine environment is a reflection of a country’s strength in the field of marine science and technology. Among the core technologies studied by USV, the problem of motion control is the ultimate goal of accomplishing its autonomous navigation mission. USV path-following is to control USV to follow a predetermined geometric path without time constraints [2]. Because there is no time limit for path-following in USV, it has great advantages for pipeline inspection, terrain tracking, area search, and other tasks. Aiming at the problem of path-following control of USV, better transient performance can be obtained by combining the guidance method with a control algorithm, thus the security of USV operation can be greatly improved [3].

The line of sight (LOS) guidance method was first applied in the field of missile flight [4]. Because it is compact, flexible, and has a wide range of applications, it has also been widely used in the USV motion control field. The LOS guidance follows a point on the desired path by mimicking the steering actions of the helmsman and controls the USV to travel to the predefined path [3]. In [5], Fossen proposed a proportional LOS (PLOS) guidance for the path-following problem and proved the control system of the method is semi-global practical finite-time stability (SGPFS). When the USV receives external disturbance, its sway velocity is not zero, resulting in a sideslip angle. The effect of wind and wave currents on the USV creates a sideslip angle and the most straightforward way to compensate for this is to gauge it by means of sensors. However, in many cases, it is difficult to measure sideslip angle accurately [6]. For this reason, the integral phase is introduced into the LOS guidance law in [7,8], and the integral LOS (ILOS) guidance method is presented to neutralize the effects of sideslip angles. An adaptive LOS (ALOS) guidance method was presented in [9]. The influence of sideslip angle on USV path-following control has been eliminated by adaptive law. In [10], a guidance method based on predictor LOS (PLOS) was proposed, which used predictor to estimate constant sideslip angle, and a USV path-following controller was devised by combining the LOS guidance method with autopilot. Ref. [11] builds on ILOS to design an adaptive headway that allows the headway to be reduced when deviating from the route in order to approach the desired path more quickly. In [7,8,9,10,11], it is presumed that the sideslip angle is constant or changes slowly. However, when the USV is disturbed by time-varying or following the curve path, the sideslip angle is time-varying [12]. Therefore, accurate estimation of time-varying sideslip angle is very important for USV path-following control. In [13], a finite-time observer was used to estimate the sideslip angle, therefore designing a path-following controller. Parers [14,15] used Time-Delay-Estimation (TDE) to estimate the time-varying sideslip angle. Ref. [16] proposed a FELOS guidance law. A filtered extended state observer was used to observe the sideslip angle caused by ocean, wind, and wave disturbances. In turn, estimates of the sideslip angle are obtained. However, in [3,12,13,14,15,16,17], it is assumed that the sideslip angle is small (less than $5^{\circ }$), and the problem of accurate measurement and estimation of large time-varying sideslip angles has not been effectively dealt with. In summary, how to perform precise USV path-following control in the absence of time-varying sideslip angle is very worthy of discussion.

Sliding mode control is a special type of variable structural control, which is strong to external disturbance, and system uncertainty has strong robustness [18]. The SMC is to lead the trajectory of the system to the selected sliding mode, and subsequently keep it in the sliding mode. The sliding control is insensitive to external disturbances and system uncertainties. The tracking error can be converted to zero in finite time by controlling the sliding variable. Paper [19] improved the USV control for the first time with SMC. However, both the above documents relied on SMC’s robustness to offset external disturbance and system uncertainty, so a large switching gain is required, which caused a large tremor of the controller and also reduced the life of the actuator. Therefore, in many cases, the adaptive control law was employed. In the above case, because the disturbance range was unknown, to avoid the excessive gain of the controller and the drastic change of the control input, a variety of adaptive finite-time convergence algorithms with self-adjusting control gain was designed [20,21,22]. Paper [23] designed an adaptive sliding mode attitude controller based on the disturbance observer, and reducing the tremor effect by the adaptive diagnosis of the perturbation observer. However, the above-mentioned operation has not considered the effects of the large sideslip angle and saturation of the actuator.

In this paper, motivated by the above considerations, a new path-following control scheme, which can estimate the large sideslip angle at a wider range of accuracy while deriving the desired heading angle, to address model uncertainties, unknown disturbances, and actuator saturation for underactuated USV. At the same time, the path-following fast non-singular terminal sliding mode (FNTSM) controller is designed to solve the problems of the underactuated USV in the existence of model uncertainties, lumped disturbances, and actuator constraints in finite-time. The key contributions of this paper can be categorized by the following points,

(1) The ELOS is designed based on the reduced-order expanded state observer. The designed ELOS guidance law cannot just derive the expected heading angle but also estimates the time-varying sideslip angle at the same time. The improved ELOS no longer places a constraint on the sideslip angle size, thus improving the accuracy of the estimate. The range of applicability of ELOS has been extended so that it can be applied to more complex environments.

(2) A fast non-singular terminal sliding mode with a faster convergence speed than the conventional non-singular terminal sliding surface is designed, and an adaptive term is introduced to update the switching term gain in real time. The proposed adaptive FNTSM not only improves the tracking accuracy and convergence speed of the USV but also reduces the actuator consumption problem caused by chattering.

(3) Considering the problem of saturation of the actuator, introducing the auxiliary dynamic system to compensate for the output saturation, and selecting appropriate design parameters. Optimization for the upper output limits that exist for the actual thrusters and servos, avoiding the generation of excessive control volumes. Improves the effectiveness of the simulation. All signals of the whole path-following closed-loop control system can be made consistent and ultimately bounded.

The remainder of this paper is structured as follows. In Section 2, preliminaries and problem formation are introduced. The guidance law based on ELOS and path following controller is designed in Section 3. The stability proof is given in Section 4. Section 5, gives the simulation studies and comparisons to explain the effectiveness of the proposed control method. Finally, the conclusions of this paper are summarized in Section 6.

## 2. Problem Formulation and Preliminaries

### 2.1. Problem Formulation

The subsection shows the model of the MSV. To facilitate the study of motion control, only its motion at the horizontal level is considered, which in turn leads to the kinematic and dynamic model of the USV as follows [24],

(1) $$\begin{array}{c} \left\{\begin{array}{c} \dot{x}=u\cos\left(\psi \right)-v\sin\left(\psi \right)\\ \dot{y}=u\sin\left(\psi \right)+v\cos\left(\psi \right)\\ \dot{\psi }=r \end{array}\right. \end{array}$$

where (x,y,ψ) represent surge position, sway position, and yaw angle of MSV concerning inertial-frame. (u,v,r), respectively, indicate the USV’s surge velocity, sway velocity, and yaw angle velocity.

With the help of the shipborne sensors, the position message (x,y), yaw angle ψ, and velocity message (u,v,r) are all measurable.

Correspondingly, the dynamic model of underactuated USV can be altered int the following way,

(2) $$\begin{array}{c} \left\{\begin{array}{c} \dot{u}=f_{u}\left(u,v,r\right)+\frac{1}{m_{11}}\tau_{u}+\frac{1}{m_{11}}d_{u}\\ \dot{v}=f_{v}\left(u,v,r\right)+\frac{1}{m_{22}}d_{v}\\ \dot{r}=f_{r}\left(u,v,r\right)+\frac{1}{m_{33}}\tau_{r}+\frac{1}{m_{33}}d_{r} \end{array}\right. \end{array}$$

where $f_{i}\left(i=u,v,r\right)$ denotes Coriolis force and centripetal force, hydrodynamic damping and the unmodelled dynamics. $\left(\tau_{u},\tau_{r}\right)$ represents the surge control force and the yaw control moment of the USV. $d_{j}\left(j=u,v,r\right)$ is the time-varying disturbance caused by the USV in the complicated marine environment. $m_{i}\left(i=11,22,33\right)$ and $d_{i}\left(i=11,22,33\right)$ are the model parameters of USV.

**Assumption** **1.** *The disturbance $d_{j}\left(j=u,v,r\right)$ is time-varying and its rate of change to the USV are bounded, satisfying*

(3) $$\begin{array}{c} \left|{\dot{d}}_{j}\right|\leq \bar{d} \end{array}$$

In practice, USV’s control input $\tau_{i}\left(i=u,r\right)$ has physical limitations. $\tau_{u}$ and $\tau_{r}$ represent the surge force and yaw moment, which are the control input of the system. The saturation function is described as,

(4) $$\begin{array}{c} sat\left(\tau_{i0}\right)=\left\{\begin{array}{c} \tau_{i\max},\;\tau_{i0}>\tau_{i\max}\\ \tau_{i0},\;\;\;\;\;\tau_{i\min}\leq \tau_{i0}\leq \tau_{i\max}\\ \tau_{i\min},\;\tau_{i0}<\tau_{i\min} \end{array}\right. \end{array}$$

where $\tau_{i0}$ is the commanded control force; $\tau_{i\max}$ and $\tau_{i\min}$ are output threshold for USV propulsion system.

Control objective: In the presence of unknown disturbances and unknown time-varying sideslip, the adaptive path following controller is designed according to the model of the USV (1) and (2) so that the USV can accurately follow the desired path $S_{d}={\left[x_{d}\left(\theta \right),y_{d}\left(\theta \right)\right]}^{T}$ without time constraints and ensures that all signals of the closed-loop control system are uniformly ultimately bounded.

### 2.2. Preliminaries

**Lemma** **1** ([25])**.** *A system considered as follows,*

(5) $$\begin{array}{c} \begin{array}{c} {\dot{\sigma }}_{0}=-\lambda_{0}L^{1/(n+1)}{\left|\sigma_{0}\right|}^{n/(n+1)}\mathrm{sgn}\left(\sigma_{0}\right)+\sigma_{1}\\ {\dot{\sigma }}_{1}=-\lambda_{1}L^{1/n}{\left|\sigma_{1}-{\dot{\sigma }}_{0}\right|}^{(n-1)/n}\mathrm{sgn}\left(\sigma_{1}-{\dot{\sigma }}_{0}\right)+\sigma_{2}\\ \vdots \\ {\dot{\sigma }}_{n-1}=-\lambda_{n-1}L^{1/2}{\left|\sigma_{n-1}-{\dot{\sigma }}_{n-2}\right|}^{1/2}\mathrm{sgn}\left(\sigma_{n-1}-{\dot{\sigma }}_{n-2}\right)+\sigma_{n}\\ {\dot{\sigma }}_{n}\in -\lambda_{n}L\mathrm{sgn}\left(\sigma_{n}-{\dot{\sigma }}_{n-1}\right)+\left[-L,L\right] \end{array} \end{array}$$

*Finite time stability. Where L and $\lambda_{i}\left(i=0,1,\cdots ,n\right)$ are both positive integers and sgn(•) is a symbolic function which is defined as follows,*

(6) $$\begin{array}{c} \mathrm{sgn}\left(x\right)=\left\{\begin{array}{c} 1\;\;\;\;\;\;\;\;\;\;\;\;\;\;\;x>0\\ 0\;\;\;\;\;\;\;\;\;\;\;\;\;\;\;x=0\\ -1\;\;\;\;\;\;\;\;\;\;\;\;x<0 \end{array}\right. \end{array}$$

*The disturbance observer designed in this way can achieve finite time convergence.*

**Lemma** **2** ([26])**.** *Consider the following Fast Non-singular Terminal Sliding surface s,*

(7) $$\begin{array}{c} s=\dot{e}+\alpha_{e}e+\beta_{e}\zeta \left(e\right) \end{array}$$

*It is noted that e is the tracking error, and $\alpha_{e}>0$, $\beta_{e}>0$ is a piecewise function. The specific design of the piecewise function ζ(e) is as follows,*

(8) $$\begin{array}{c} \zeta \left(e\right)=\left\{\begin{array}{c} sig^{a}\left(e\right),\;\;\;\;\;\;\;\;\;\;\;\;\;\;\;\;\;\;\bar{s}=0\;or\;\left(\bar{s}\neq 0\;and\;\left|e\right|>\phi \right)\\ r_{1}e+r_{2}sig^{2}\left(e\right),\;\;\;\;\;\;\bar{s}\neq 0\;and\;\left|e\right|\leq \phi \end{array}\right. \end{array}$$

*where $sig^{\gamma }\left(x\right)={\left|x\right|}^{\gamma }sgn\left(x\right)$, $\bar{s}=\dot{e}+\alpha_{e}e+\beta_{e}sig^{a}\left(e\right)$, 0<a, $r_{1}=\left(2-a\right)\phi^{a-1}$, $r_{2}=\left(a\;-\;1\right)\phi^{a-2}$, and ϕ is a small constant. When the sliding mode surface s enters the sliding state, the tracking error can converge to zero in finite time.*

**Lemma** **3** ([27])**.** *To design an adaptive switching term, consider the typical first-order sliding-mode dynamic equation as follow,*

(9) $$\begin{array}{c} \dot{\varsigma }\left(t\right)=d\left(t\right)+u\left(t\right) \end{array}$$

*Among them, ς(t)∈R represents the sliding mode surface that is affected by the switching function and reaches the origin within a finite time, u(t) represents the control input and d(t) represents the uncertainty. If d(t) considers that its first derivative and its second derivative are bounded to satisfy $\left|d(t)\right|<d_{0}$, $\left|\dot{d}\left(t\right)\right|<d_{1}$, $\left|\ddot{d}\left(t\right)\right|<d_{2}$. Consider the control input as,*

(10) $$\begin{array}{c} u(t)=-(k(t)+\eta )\mathrm{sgn}(\varsigma (t)) \end{array}$$

*where η is a normal number, k(t) is a variable term.*

*(1) When the upper bound $d_{0}$ is unknown and $d_{1}$ is known k(t) can be updated by the following two adaptive laws,*

(11) $$\begin{array}{c} \dot{k}\left(t\right)=-\rho \left(t\right)\mathrm{sgn}\left(\delta \left(t\right)\right) \end{array}$$

(12) $$\begin{array}{c} \dot{r}\left(t\right)=\gamma \left|\delta (t)\right|+r_{0}\sqrt{\gamma }\mathrm{sgn}\left(e\left(t\right)\right) \end{array}$$

*where $\rho \left(t\right)=r_{0}+r\left(t\right)$, $\delta \left(t\right)=k\left(t\right)-\frac{1}{\alpha }\left|\dot{\bar{u_{eq}}}\left(t\right)\right|-\varepsilon$, $e\left(t\right)=q\frac{d_{1}}{\alpha }-r\left(t\right)$, ${\dot{\bar{u}}}_{eq}\left(t\right)=\frac{1}{\tau }\left(u\left(t\right)-{\bar{u}}_{eq}\left(t\right)\right)$, 0<α<1, γ, $r_{0}$, ε are all positive constant, τ is a very small time constant. To ensure $\left|{\bar{u}}_{eq}+d\left(t\right)\right|$ as small as possible, q>sup(1,ddt(u¯eq(t))ddt(u¯eq(t))d1d1) is safety margin, sup is the minimum upper bound function, k(t) can reach $k\left(t\right)>d_{0}$ within a limited time, to ensure that the sliding surface is maintained in a sliding state. In addition, the gain k(t) and ρ(t) is bounded.*

*(2) When the upper bounds $d_{0}$ and $d_{1}$ are both unknown, however the upper bound of the second derivative $d_{2}$ is known, k(t) can be updated by the following two layers of adaptive laws,*

(13) $$\begin{array}{c} \dot{k}\left(t\right)=-\rho \left(t\right)\mathrm{sgn}\left(\delta \left(t\right)\right) \end{array}$$

(14) $$\begin{array}{c} \dot{r}\left(t\right)=\left\{\begin{array}{c} \gamma \left|\delta (t)\right|,\;\;\left|\delta (t)\right|>\delta_{0}\\ 0,\;\;\;\;\;\;\;\;\;\;\;\;\left|\delta (t)\right|\leq \delta_{0} \end{array}\right. \end{array}$$

*where $\rho \left(t\right)=r_{0}+r\left(t\right)$, $\delta \left(t\right)=k\left(t\right)-\frac{1}{\alpha }\left|{\bar{u}}_{eq}\left(t\right)\right|-\varepsilon$, ${\dot{\bar{u}}}_{eq}\left(t\right)=\frac{1}{\tau }\left(u\left(t\right)-{\bar{u}}_{eq}\left(t\right)\right)$, 0<α<1, γ, $r_{0}$, ε are all positive constant. In particular, q>sup(1,ddt(u¯eq(t))ddt(u¯eq(t))d2d2) satisfy the following inequality,*

(15) $$\begin{array}{c} \frac{1}{4}\varepsilon^{2}>\delta_{0}^{2}+\frac{1}{\gamma }{\left(\frac{qd_{2}}{\alpha }\right)}^{2} \end{array}$$

*The gain k(t) can reach $k\left(t\right)>d_{0}$ in a limited time to ensure a continuous sliding state. In addition, the gain k(t) and ρ(t) is bounded.*

**Remark** **1.** *It is known that (9) is not required to be the complete dynamics of the controlled object; however it represents the dynamics of the sliding variable. After the compensated dynamics, the Lemma3 still holds.*

## 3. Path following Control

### 3.1. Elos Guidance Law Design

**Figure 1:**
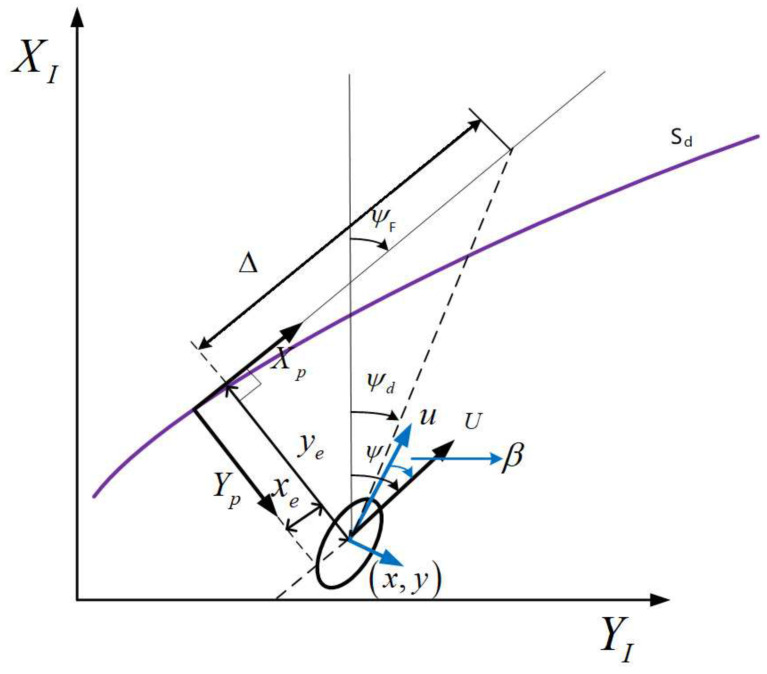
Schematic diagram of USV path-following guidance.

For a USV in Figure 1 located at the coordinate point (x,y), its position error ${\left[x_{e},y_{e}\right]}^{T}$ relative to the desired path $S_{d}={\left[x_{d}\left(\theta \right),y_{d}\left(\theta \right)\right]}^{T}$ can be expressed as,

(16) $$\left[\begin{array}{c} x_{e}\\ y_{e} \end{array}\right]=\left[\begin{array}{cc} \cos\psi_{F} & \sin\psi_{F}\\ -\sin\psi_{F} & \cos\psi_{F} \end{array}\right]\left[\begin{array}{c} x-x_{d}\left(\theta \right)\\ y-y_{d}\left(\theta \right) \end{array}\right]$$

Derivation of the above formula can be obtained,

(17) $$\begin{array}{c} \left\{\begin{array}{c} {\dot{x}}_{e}=u\cos\left(\psi -\psi_{F}\right)-u\sin\left(\psi -\psi_{F}\right)\tan\beta +{\dot{\psi }}_{F}y_{e}-u_{p}\\ {\dot{y}}_{e}=u\sin\left(\psi -\psi_{F}\right)+u\cos\left(\psi -\psi_{F}\right)\tan\beta -{\dot{\psi }}_{F}x_{e} \end{array}\right. \end{array}$$

where the sideslip angle is β=atan2(v,u) and the speed of the virtual reference point is $u_{p}=\dot{\theta }\sqrt{x_{d}^{\prime }{}^{2}\left(\theta \right)+y_{d}^{\prime }{}^{2}\left(\theta \right)}$ which can be seen as a control input to control the convergence of the longitudinal tracking error $x_{e}$.

**Remark** **2.** *In most of the literature, the sideslip angle β is assumed to be small (The sideslip angle is usually assumed to be less than $5^{\circ }$) [5,7,13,14,17,25,28], so that the conditions sinβ≈β and cosβ≈1 hold. However, the premise of this article is that the sideslip angle is large, and the above assumption is not true. In the case of high lateral disturbances, the USV is subject to sideslip angles greater than $10^{\circ }$ caused by the disturbance of wind and wave currents. It is worth noting that the small-angle approximation principle increases the error by an order of magnitude at $12^{\circ }$ and $18^{\circ }$, respectively.*

The horizontal error can be sorted out,

(18) $$\begin{array}{c} y_{e}=u\sin\left(\psi -\psi_{F}\right)+g-{\dot{\psi }}_{F}x_{e} \end{array}$$

where $g=u\cos(\psi -\psi_{F})\tan\beta$. The design reduced-order ESO estimate *g* contains unknown terms β, and its expression is

(19) $$\begin{array}{c} \left\{\begin{array}{c} \dot{p}=-kp-k^{2}y_{e}-k\left[u\sin\left(\psi -\psi_{F}\right)-{\dot{\psi }}_{F}x_{e}\right]\\ \hat{g}=p+ky_{e} \end{array}\right. \end{array}$$

Among them, *p* represents the auxiliary state of the observer, *k* is the design parameter of the observer. Since $u\cos(\psi -\psi_{F})$ is known, the estimated value of sideslip angle $\hat{\beta }$ can be obtained as,

(20) $$\begin{array}{c} \hat{\beta }=\arctan\left(\frac{\hat{g}}{u\cos(\psi -\psi_{F})}\right) \end{array}$$

Define the estimated error of the reduced-order ESO as $\tilde{g}=g-\hat{g}$. Take the derivative of $\tilde{g}$ and insert Equations (18) and (19) to obtain,

(21) $$\begin{array}{cc} \dot{\tilde{g}} & =\dot{g}-\dot{p}-k{\dot{y}}_{e}\\ \; & =\dot{g}+kp+k^{2}y_{e}+k\left[u\sin\left(\psi -\psi_{F}\right)-{\dot{\psi }}_{F}x_{e}\right]-k\left[u\sin\left(\psi -\psi_{F}\right)+g-{\dot{\psi }}_{F}x_{e}\right]\\ \; & =\dot{g}-k\tilde{g} \end{array}$$

**Assumption** **2.** *The rate of changing of the unknown term g is bounded, which satisfies $\left|\dot{g}\right|\leq \bar{g}$ and $\bar{g}$ is a normal number.*

**Lemma** **4.** *Under the condition of Assumption 2, by increasing the bandwidth of ESO, the estimation error $\tilde{g}$ can converge to ϖϖkk in max(0,lnklnkkk), where ϖ is a positive number.*

For the detailed proof of Lemma 4, Section 2 of [29] gives detailed proof.

To obtain the ideal heading angle, the design guidance law is

(22) $$\begin{array}{c} \psi_{d}=\psi_{F}+\arctan\left(-\frac{y_{e}}{\Delta }-\tan\hat{\beta }\right) \end{array}$$

To converge the longitudinal tracking error $x_{e}$, design the velocity $u_{p}$ of the virtual reference point of the desired path,

(23) $$\begin{array}{c} u_{p}=u\cos\left(\psi -\psi_{F}\right)-u\sin\left(\psi -\psi_{F}\right)\tan\hat{\beta }+k_{s}x_{e} \end{array}$$

Then the updated law of path parameters θ can be obtained as,

(24) $$\begin{array}{c} \dot{\theta }=\frac{u_{p}}{\sqrt{x_{d}^{\prime }{}^{2}\left(\theta \right)+y_{d}^{\prime }{}^{2}\left(\theta \right)}} \end{array}$$

**Assumption** **3.** *The ideal heading angle $\psi_{d}$ given by the guidance system can be accurately tracked by the dynamics controller, namely $\psi -\psi_{d}=0$.*

According to Assumption 3 and Formula (22),

(25) $$\begin{array}{c} \left\{\begin{array}{c} \sin\left(\arctan\left(-\frac{y_{e}}{\Delta }-\tan\beta \right)\right)=-\frac{y_{e}+\Delta \tan\hat{\beta }}{\sqrt{\Delta^{2}+{\left(y_{e}+\Delta \hat{\beta }\right)}^{2}}}\\ \cos\left(\arctan\left(-\frac{y_{e}}{\Delta }-\tan\beta \right)\right)=\frac{\Delta }{\sqrt{\Delta^{2}+{\left(y_{e}+\Delta \hat{\beta }\right)}^{2}}} \end{array}\right. \end{array}$$

Substituting Equations (23) and (25) into Equation (17), we can obtain

(26) $$\begin{array}{c} \left\{\begin{array}{c} {\dot{x}}_{e}=-k_{s}x_{e}+\psi_{F}y_{e}-u\sin\left(\psi -\psi_{F}\right)\left(\tan\beta -\tan\hat{\beta }\right)\\ {\dot{y}}_{e}=-Cy_{e}-\psi_{F}x_{e}+\Delta C_{1}\left(\tan\beta -\tan\hat{\beta }\right) \end{array}\right. \end{array}$$

where C1=u/uΔ2+(ye+Δtanβ^)2Δ2+(ye+Δtanβ^)2.

According to Lemma 4, we know $(\tan\beta -\tan\hat{\beta })\approx 0$. Design Lyapunov function for guidance system,

(27) $$\begin{array}{c} V_{1}=\frac{1}{2}\left(x_{e}^{2}+y_{e}^{2}+{\tilde{g}}^{2}\right) \end{array}$$

Derivation of the above formula and substituting Formulas (21) and (26) to obtain,

(28) $$\begin{array}{c} {\dot{V}}_{1}=-k_{s}x_{e}^{2}-C_{1}y_{e}^{2}-k{\tilde{g}}^{2}+\dot{g}\tilde{g} \end{array}$$

### 3.2. Path following Controller Design

**Figure 2:**
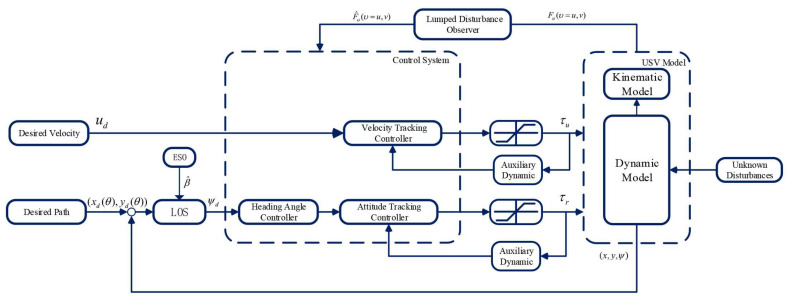
The Block Diagram of The Path Following Controller.

In this part, first, a finite-time disturbance observer is designed to accurately estimate the external disturbance and the perturbation parameter. Then, in order to track the yaw angle $\psi_{d}$ and forward velocity $u_{d}$, the attitude tracking controller and the velocity tracking controller are designed based on the fast non-singular terminal sliding mode. The introduction of the auxiliary power system solves the problem of saturation of the actuator during the actual heading. The block diagram of the proposed controller is shown in Figure 2.

#### 3.2.1. Design of the Finite-Time Lumped Disturbance Observer

Consider the under-driven unmanned ship model with lumped disturbances as follows,

(29) $$\begin{array}{c} \left\{\begin{array}{c} m_{11}\dot{u}=F_{u}\left(u,v,r\right)+\tau_{u}\\ m_{22}\dot{v}=F_{v}\left(u,v,r\right)\\ m_{33}\dot{r}=F_{r}\left(u,v,r\right)+\tau_{r} \end{array}\right. \end{array}$$

where $F_{u}=m_{11}f_{u}+d_{u}$, $F_{v}=m_{22}f_{v}+d_{v}$, $F_{r}=m_{33}f_{r}+d_{r}$.

The finite-time lumped disturbance observer is designed as follows,

(30) $$\begin{array}{c} \begin{array}{c} M\dot{\hat{\nu }}=\Lambda +\tau \\ \Lambda =-\lambda_{1}L^{\frac{1}{2}}sig^{\frac{1}{2}}\left(M\hat{\nu }-M\nu \right)+{\hat{F}}_{\nu }\\ {\dot{\hat{F}}}_{\nu }=-\lambda_{2}Lsign\left({\hat{F}}_{\nu }-\Lambda \right) \end{array} \end{array}$$

where $M=\left[\begin{array}{ccc} m_{11} & 0 & 0\\ 0 & m_{22} & 0\\ 0 & 0 & m_{33} \end{array}\right]$, $\nu =[u,v,r{]}^{T}$, $\Lambda =[\Lambda_{u},\Lambda_{v},\Lambda_{r}{]}^{T}$, $L=\mathrm{diag}(l_{1},l_{2})>0$, $\lambda_{1}>0$, $\lambda_{2}>0$.

**Theorem** **1.** *Based on the designed finite-time disturbance observer, the unknown external disturbance ${\hat{\tau }}_{d}$ can be accurately estimated within a finite time.*

**Proof.**  The definition error is as follows,

(31) $$\begin{array}{cc} M\dot{\tilde{\nu }} & =-\lambda_{1}L^{\frac{1}{2}}sig^{\frac{1}{2}}\left(M\tilde{\nu }\right)+{\hat{F}}_{\nu }+\tau -M\dot{v}\\ \; & =-\lambda_{1}L^{\frac{1}{2}}sig^{\frac{1}{2}}\left(M\tilde{\nu }\right)+{\tilde{F}}_{\nu } \end{array}$$

(32) $$\begin{array}{cc} {\dot{\tilde{F}}}_{\nu } & =-\lambda_{2}Lsign\left({\hat{F}}_{\nu }-\Lambda \right)-F_{\nu }\\ \; & \in -\lambda_{2}Lsign\left(M\tilde{\nu }\right)+\left[-D,D\right] \end{array}$$

where $\tilde{\nu }=\nu -\hat{\nu }$, ${\tilde{F}}_{\nu }=F_{\nu }-{\hat{F}}_{\nu }$. Applying Lemma 1, it can be concluded that the error of the finite-time disturbance observer can converge to zero, i.e., there is a finite time $T_{0}$ so that,

(33) $$\begin{array}{c} \hat{\nu }\left(t\right)\equiv \nu \left(t\right),{\hat{F}}_{\nu }\equiv F_{\nu },\forall t\geq T_{0} \end{array}$$

□

#### 3.2.2. Attitude Tracking Controller Design

Define the heading angle tracking error $\psi_{e}$ as,

(34) $$\begin{array}{c} \psi_{e}=\psi -\psi_{d} \end{array}$$

Then derivation of the $\psi_{e}$ can be obtained,

(35) $$\begin{array}{c} {\dot{\psi }}_{e}=r-{\dot{\psi }}_{d} \end{array}$$

Design of fast non-singular terminal sliding surface $s_{\psi }$ for heading angle error as,

(36) $$\begin{array}{c} s_{\psi }={\dot{\psi }}_{e}+\alpha_{\psi }\psi_{e}+\beta_{\psi }\zeta \left(\psi_{e}\right) \end{array}$$

where $\alpha_{\psi }>0$, $\beta_{\psi }>0$. The specific design of the piecewise function $\zeta (\psi_{e})$ is as follows,

(37) $$\begin{array}{c} \zeta \left(\psi_{e}\right)=\left\{\begin{array}{c} sig^{a}\left(\psi_{e}\right),\;\;\;\;\;\;\;\;\;\;\;\;\;\;\;\;\;\;\;\;{\bar{s}}_{\psi }=0\;or\;\left({\bar{s}}_{\psi }\neq 0\;and\;\left|\psi_{e}\right|>\phi \right)\\ r_{\psi 1}\psi_{e}+r_{\psi 2}sig^{2}\left(\psi_{e}\right),\;\;{\bar{s}}_{\psi }\neq 0\;and\;\left|\psi_{e}\right|\leq \phi \end{array}\right. \end{array}$$

where ${\bar{s}}_{\psi }={\dot{\psi }}_{e}+\alpha_{\psi }\psi_{e}+\beta_{\psi }sig^{a}\left(\psi_{e}\right)$, $0<a_{\psi }$, $r_{\psi 1}=\left(2-a\right)\phi^{a-1}$, $r_{\psi 2}=\left(a-1\right)\phi^{a-2}$, ϕ is a small positive constant. Continue to derive the $s_{\psi }$,

(38) $$\begin{array}{c} {\dot{s}}_{\psi }={\ddot{\psi }}_{e}+\alpha_{\psi }{\dot{\psi }}_{e}+\beta_{\psi }\dot{\zeta }\left(\psi_{e}\right) \end{array}$$

where $\dot{\zeta }\left(\psi_{e}\right)$ expressed as,

(39) $$\begin{array}{c} \dot{\zeta }\left(\psi_{e}\right)=\left\{\begin{array}{c} a{\left|\psi_{e}\right|}^{a-1}{\dot{\psi }}_{e},\;\;\;\;\;\;\;\;\;\;\;\;{\bar{s}}_{\psi }=0\;or\;\left({\bar{s}}_{\psi }\neq 0,\left|\psi_{e}\right|>\phi \right)\\ r_{1}{\dot{\psi }}_{e}+2r_{2}\left|\psi_{e}\right|{\dot{\psi }}_{e},\;\;{\bar{s}}_{\psi }\neq 0\;and\;\left|\psi_{e}\right|\leq \phi \end{array}\right. \end{array}$$

Based on the above analysis, the adaptive synovial heading tracking control law $\tau_{r}$ is designed as follows,

(40) $$\begin{array}{c} \tau_{r}=-m_{33}\left(\frac{{\hat{F}}_{r}}{m_{33}}-{\ddot{\psi }}_{d}+\alpha_{\psi }{\dot{\psi }}_{e}+\beta_{\psi }\dot{\zeta }\left(\psi_{e}\right)\right)-m_{33}\left(\eta_{r}+k_{r}\left(t\right)\right)s_{\psi } \end{array}$$

Among them, the introduced adaptive term updates the switching term gain $k_{r}\left(t\right)$ in real time, and its adaptive law is updated in the following form,

(41) $$\begin{array}{c} \left\{\begin{array}{c} {\dot{k}}_{r}\left(t\right)=-\rho_{r}\left(t\right)\mathrm{sgn}\left(\delta_{r}\left(t\right)\right)\\ {\dot{r}}_{r}\left(t\right)=\gamma_{r}\left|\delta_{r}\left(t\right)\right|+r_{0,r}\sqrt{\gamma_{r}}\mathrm{sgn}\left({\tilde{e}}_{r}\left(t\right)\right) \end{array}\right. \end{array}$$

where $\gamma_{r}$, $r_{0,r}>0$, $\rho_{r}\left(t\right)=r_{0,r}+r_{r}\left(t\right)$, $\delta_{r}\left(t\right)=k_{r}\left(t\right)-\frac{1}{\kappa_{r}}\left|{\bar{u}}_{eq,r}\left(t\right)\right|-\varepsilon_{r}$, ${\tilde{e}}_{r}\left(t\right)=q_{r}\frac{d_{2,r}}{\kappa_{r}}-r_{r}\left(t\right)$, ${\dot{\bar{u}}}_{eq,r}\left(t\right)=\frac{1}{\mu_{r}}\left(\left(-\left(\eta_{r}+k_{r}\left(t\right)\right)\mathrm{sgn}\left(s_{\psi }\right)\right)-{\bar{u}}_{eq,r}\left(t\right)\right)$, qr>sup(1,ddt(u¯eq,r(t))ddt(u¯eq,r(t))Frd2,r), $0\;<\;\kappa_{r}\;<\;1$, $\varepsilon_{r}$, $\mu_{r}>0$, $d_{2,r}=k_{d,r}\left(L_{1}+\left|{\hat{d}}_{r}\right|\right)+L_{2}$. At the same time, considering the input saturation problem existing in the actual situation of the unmanned ship, the following form of an auxiliary dynamic system is introduced to compensate for the saturation of the system output,

(42) $$\begin{array}{c} {\dot{e}}_{r}=\left\{\begin{array}{c} -K_{er}e_{r}-\frac{\left|S_{\psi }\cdot \Delta \tau_{r}\right|+0.5\Delta \tau_{r}^{2}}{e_{r}}+\Delta \tau_{r},\left|e_{r}\right|\geq \xi_{r}\\ 0,\;\;\;\;\;\;\;\;\;\;\;\;\;\;\;\;\;\;\;\;\;\;\;\;\;\;\;\;\;\;\;\;\;\;\;\;\;\;\;\;\;\;\;\;\;\left|e_{r}\right|<\xi_{r} \end{array}\right. \end{array}$$

The designed control law for the yaw angle subsystem is

(43) $$\begin{array}{c} \tau_{r0}=-m_{33}\left(\frac{{\hat{F}}_{r}}{m_{33}}-{\ddot{\psi }}_{d}+\alpha_{\psi }{\dot{\psi }}_{e}+\beta_{\psi }\dot{\zeta }\left(\psi_{e}\right)\right)-m_{33}\left(\eta_{r}+k_{r}\left(t\right)\right)s_{\psi }+K_{r}e_{r} \end{array}$$

Design the Lyapunov function as follows,

(44) $$\begin{array}{c} V_{\psi }=\frac{m_{33}}{2}s_{\psi }^{2}+\frac{1}{2}e_{r}^{2} \end{array}$$

Derivation of the $V_{\psi }$, and Formulas (38), (42) and (43) into it to obtain

(45) $$\begin{array}{cc} {\dot{V}}_{\psi } & =m_{33}s_{\psi }{\dot{s}}_{\psi }+e_{r}{\dot{e}}_{r}\\ & =m_{33}s_{\psi }\left({\ddot{\psi }}_{e}+\alpha_{\psi }{\dot{\psi }}_{e}+\beta_{\psi }\dot{\zeta }\left(\psi_{e}\right)\right)+e_{r}{\dot{e}}_{r}\\ & =m_{33}s_{\psi }\left(\dot{r}-{\ddot{\psi }}_{d}+\alpha_{\psi }{\dot{\psi }}_{e}+\beta_{\psi }\dot{\zeta }\left({\dot{\psi }}_{e}\right)\right)+e_{r}{\dot{e}}_{r}\\ & =m_{33}s_{\psi }\left[\left(f_{r}+\frac{1}{m_{33}}\left(\tau_{r}+\Delta \tau_{r}\right)+\frac{1}{m_{33}}d_{r}-{\ddot{\psi }}_{d}\right)+\alpha_{\psi }{\dot{\psi }}_{e}+\beta_{\psi }\dot{\zeta }\left({\dot{\psi }}_{e}\right)\right]+e_{r}{\dot{e}}_{r}\\ & =m_{33}s_{\psi }\left[\frac{1}{m_{33}}(-m_{33}\left(\alpha_{\psi }{\dot{\psi }}_{e}+\beta_{\psi }\dot{\zeta }\left({\dot{\psi }}_{e}\right)\right)-m_{33}\left(\eta_{r}+k_{r}\left(t\right)\right)s_{\psi }+K_{r}e_{r}+\Delta \tau_{r}(+\alpha_{\psi }{\dot{\psi }}_{e}+\beta_{\psi }\dot{\zeta }\left(\psi_{e}\right)\right]+e_{r}{\dot{e}}_{r}\\ & =s_{\psi }\left[-m_{33}\left(\eta_{r}+k_{r}\left(t\right)\right)s_{\psi }+K_{r}e_{r}+\Delta \tau_{r}\right]-\left|s_{\psi }\cdot \Delta \tau_{r}\right|-0.5\Delta \tau_{r}^{2}+e_{r}\Delta \tau_{r}\\ & =-\left(\eta_{r}+k_{r}\left(t\right)\right)m_{33}s_{\psi }^{2}+K_{r}s_{\psi }e_{r}+s_{\psi }\Delta \tau_{r}-K_{er}{e^{2}}_{r}-\left|s_{\psi }\cdot \Delta \tau_{r}\right|-0.5\Delta \tau_{r}^{2}+e_{r}\Delta \tau_{r} \end{array}$$

According to Lemma 3, the designed control law uses double-layer adaptive law (41), which makes $k_{r}>\left|d_{r}\right|$ true in a limited time, and to ensure $\rho_{r}$ and $k_{r}$ are bound. Therefore, the Formula (45) satisfies,

(46) $$\begin{array}{c} {\dot{V}}_{\psi }\leq -\eta_{r}m_{33}{s_{\psi }}^{2}+K_{r}s_{\psi }e_{r}+s_{\psi }\Delta \tau_{r}-K_{er}{e^{2}}_{r}-\left|s_{\psi }\cdot \Delta \tau_{r}\right|-0.5\Delta {\tau_{r}}^{2}+e_{r}\Delta \tau_{r} \end{array}$$

According to Young’s inequality, there are

(47) $$\begin{array}{c} K_{r}s_{\psi }e_{r}\leq \frac{1}{2}K_{r}{s_{\psi }}^{2}+\frac{1}{2}K_{r}{e_{r}}^{2},e_{r}\Delta \tau_{r}\leq \frac{1}{2}{e_{r}}^{2}+\frac{1}{2}\Delta {\tau_{r}}^{2} \end{array}$$

Applying the above inequality, Equation (46) becomes

(48) $$\begin{array}{cc} {\dot{V}}_{\psi } & \leq -\eta_{r}m_{33}{s_{\psi }}^{2}+\frac{1}{2}K_{r}{s_{\psi }}^{2}+\frac{1}{2}K_{r}{e_{r}}^{2}-K_{er}{e^{2}}_{r}-0.5\Delta {\tau_{r}}^{2}+\frac{1}{2}{e_{r}}^{2}+\frac{1}{2}\Delta {\tau_{r}}^{2}\\ \; & \leq -\left(\eta_{r}-\frac{1}{2}K_{r}\right)m_{33}{s_{\psi }}^{2}-\left(K_{er}-\frac{1}{2}K_{r}-\frac{1}{2}\right){e^{2}}_{r} \end{array}$$

#### 3.2.3. Velocity Tracking Controller Design

Define the desired forward velocity as $u_{d}$, so the velocity tracking error $u_{e}$ can be obtained as

(49) $$\begin{array}{c} u_{e}=u-u_{d} \end{array}$$

Taking the derivative of the above formula, we can obtain

(50) $$\begin{array}{c} {\dot{u}}_{e}=\dot{u}-{\dot{u}}_{d} \end{array}$$

Design of fast non-singular terminal sliding surface $s_{u}$ for heading angle error,

(51) $$\begin{array}{c} s_{u}={\dot{u}}_{e}+\alpha_{u}u_{e}+\beta_{u}\zeta \left(u_{e}\right) \end{array}$$

where $\alpha_{u}>0$, $\beta_{u}>0$. The specific design for the piecewise function $\zeta (u_{e})$ is as follows,

(52) $$\begin{array}{c} \zeta \left(u_{e}\right)=\left\{\begin{array}{c} sig^{a}\left(u_{e}\right),\;\;\;\;\;\;\;\;\;\;\;\;\;\;\;\;\;\;\;{\bar{s}}_{u}=0\;or\;\left({\bar{s}}_{u}\neq 0\;and\;\left|u_{e}\right|>\phi \right)\\ r_{u1}u_{e}+r_{u2}sig^{2}\left(u_{e}\right),\;\;{\bar{s}}_{u}\neq 0\;and\;\left|u_{e}\right|\leq \phi \end{array}\right. \end{array}$$

where ${\bar{s}}_{u}={\dot{u}}_{e}+\alpha_{u}u_{e}+\beta_{u}sig^{a}\left(u_{e}\right)$, 0<a, $r_{u1}=\left(2-a\right)\phi^{a-1}$, $r_{u2}=\left(a-1\right)\phi^{a-2}$, ϕ is a small constant. Continue to derive $s_{u}$,

(53) $$\begin{array}{c} {\dot{s}}_{u}={\dot{u}}_{e}+\alpha_{u}u_{e}+\beta_{u}\dot{\zeta }\left(u_{e}\right) \end{array}$$

where $\dot{\zeta }\left(u_{e}\right)$ mains,

(54) $$\begin{array}{c} \dot{\zeta }\left(u_{e}\right)=\left\{\begin{array}{c} a{\left|u_{e}\right|}^{a-1}{\dot{u}}_{e},\;\;\;\;\;\;\;\;\;\;\;\;\;\;\;{\bar{s}}_{u}=0\;or\;\left({\bar{s}}_{u}\neq 0\;and\;\left|u_{e}\right|>\phi \right)\\ r_{u1}{\dot{u}}_{e}+2r_{u2}\left|u_{e}\right|{\dot{u}}_{e},\;\;{\bar{s}}_{u}\neq 0\;and\;\left|u_{e}\right|\leq \phi \end{array}\right. \end{array}$$

Based on the above analysis, design the adaptive synovial heading tracking control law $\tau_{u}$ as

(55) $$\begin{array}{c} \tau_{u}=-m_{11}\left(\frac{{\hat{F}}_{u}}{m_{11}}-{\dot{u}}_{d}-\frac{-{\ddot{u}}_{e}-\beta_{u}\dot{\zeta }\left(u_{e}\right)}{\alpha_{u}}\right)-m_{11}\left(\eta_{u}+k_{u}\left(t\right)\right)s_{u} \end{array}$$

where $k_{u}\left(t\right)$ is the introduction of the adaptive term to update the gain of the switching term in real time. The adaptive law is updated in the following form,

(56) $$\begin{array}{c} \left\{\begin{array}{c} {\dot{k}}_{u}\left(t\right)=-\rho_{u}\left(t\right)\mathrm{sgn}\left(\delta_{u}\left(t\right)\right)\\ {\dot{r}}_{u}\left(t\right)=\gamma_{u}\left|\delta_{u}\left(t\right)\right|+r_{0,u}\sqrt{\gamma_{u}}\mathrm{sgn}\left({\tilde{e}}_{u}\left(t\right)\right) \end{array}\right. \end{array}$$

where $\gamma_{u}$, $r_{0,u}>0$, $\rho_{u}\left(t\right)=r_{0,u}+r_{u}\left(t\right)$, $\delta_{u}\left(t\right)=k_{u}\left(t\right)-\frac{1}{\kappa_{u}}\left|{\bar{u}}_{eq,u}\left(t\right)\right|-\varepsilon_{u}$, ${\tilde{e}}_{u}\left(t\right)=q_{u}\frac{d_{2,u}}{\kappa_{u}}-$$r_{u}\left(t\right)$, ${\dot{\bar{u}}}_{eq,u}\left(t\right)=\frac{1}{\mu_{u}}\left(\left(-\left(\eta_{u}+k_{u}\left(t\right)\right)\mathrm{sgn}\left(s_{u}\right)\right)-{\bar{u}}_{eq,u}\left(t\right)\right)$, $0<\kappa_{u}<1$, $\varepsilon_{u}$, $\mu_{u}>0$, $d_{2,u}=k_{d,u}\left(L_{1}+\left|{\hat{d}}_{u}\right|\right)+L_{2}$, qu>sup(1,ddt(u¯eq,u(t))ddt(u¯eq,u(t))Fu/d2,u).

At the same time, the following forms of auxiliary dynamic systems are introduced,

(57) $$\begin{array}{c} {\dot{e}}_{u}=\left\{\begin{array}{c} -K_{eu}e_{u}-\frac{\left|s_{u}\cdot \Delta \tau_{u}\right|+0.5\Delta \tau_{u}^{2}}{e_{u}}+\Delta \tau_{u},\;\left|e_{u}\right|\geq \xi_{u}\\ 0,\;\;\;\;\;\;\;\;\;\;\;\;\;\;\;\;\;\;\;\;\;\;\;\;\;\;\;\;\;\;\;\;\;\;\;\;\;\;\;\;\;\;\;\;\;\;\;\;\left|e_{u}\right|<\xi_{u} \end{array}\right. \end{array}$$

Then the input instruction for the control of the surge velocity can be designed as follows,

(58) $$\begin{array}{c} \tau_{u0}=-m_{11}\left(\frac{{\hat{F}}_{u}}{m_{11}}-{\dot{u}}_{d}-\frac{-{\ddot{u}}_{e}-\beta_{u}\dot{\zeta }\left(u_{e}\right)}{\alpha_{u}}\right)-m_{11}\left(\eta_{u}+k_{u}\left(t\right)\right)s_{u}+K_{u}e_{u} \end{array}$$

Design the Lyapunov function as follows,

(59) $$\begin{array}{c} V_{u}=\frac{m_{11}}{2}\alpha_{u}s_{u}^{2}+\frac{1}{2}e_{u}^{2} \end{array}$$

Derivation of the $V_{u}$, and put Formula (53), (57) and (58) into (59),

(60) $$\begin{array}{cc} {\dot{V}}_{u} & =m_{11}\alpha_{u}s_{u}{\dot{s}}_{u}+e_{u}{\dot{e}}_{u}\\ & =-\left(\eta_{u}+k_{u}\left(t\right)\right)m_{11}s_{u}^{2}+K_{u}s_{u}e_{u}+s_{u}\Delta \tau_{u}-K_{eu}{e^{2}}_{u}-\left|s_{u}\cdot \Delta \tau_{u}\right|-0.5\Delta \tau_{u}^{2}+e_{u}\Delta \tau_{u} \end{array}$$

According to Lemma 3, the designed control law uses the double-layer adaptive law (56), which makes $k_{u}>\left|d_{u}\right|$ in a finite time, and guarantees $\rho_{u}$, $k_{u}$ bounded. Therefore, the Formula (60) satisfies,

(61) $$\begin{array}{c} {\dot{V}}_{u}\leq -m_{11}\eta_{u}{s_{u}}^{2}+K_{r}s_{u}e_{u}+s_{u}\Delta \tau_{u}-K_{eu}{e^{2}}_{u}-\left|s_{u}\cdot \Delta \tau_{u}\right|-0.5\Delta {\tau_{u}}^{2}+e_{u}\Delta \tau_{u} \end{array}$$

According to Young’s inequality, there are,

(62) $$\begin{array}{c} K_{u}s_{u}e_{u}\leq \frac{1}{2}K_{u}{s_{u}}^{2}+\frac{1}{2}K_{u}{e_{u}}^{2},e_{u}\Delta \tau_{u}\leq \frac{1}{2}{e_{u}}^{2}+\frac{1}{2}\Delta {\tau_{u}}^{2} \end{array}$$

Applying the above inequality, Equation (61) becomes

(63) $$\begin{array}{cc} {\dot{V}}_{u} & \leq -m_{11}\eta_{u}{s_{u}}^{2}+K_{r}s_{u}e_{u}+s_{u}\Delta \tau_{u}-K_{eu}{e^{2}}_{u}-\left|s_{u}\cdot \Delta \tau_{u}\right|-0.5\Delta {\tau_{u}}^{2}+e_{u}\Delta \tau_{u}\\ \; & \leq -\left(\eta_{u}-\frac{1}{2}K_{u}\right)m_{11}{s_{u}}^{2}-\left(K_{eu}-\frac{1}{2}K_{u}-\frac{1}{2}\right){e^{2}}_{u} \end{array}$$

## 4. Stability Analysis

**Theorem** **2.** *With Assumptions 1 to 3, for the USV mathematical models (12) and (13), the design is based on the reduced-order ESO (19) for the interference of unknown time-varying disturbances and the existence of time-varying large sideslip angle. Under the condition of the ELOS guidance law (22), parameter adaptive update law (24), design an adaptive fast non-singular terminal sliding mode control law (43) and (58), based on finite time disturbance observer (30) along with the auxiliary dynamic systems (42) and (57), and by selecting appropriate parameters, all signals of the path-following closed-loop control system can be made uniformly ultimately bounded.*

**Proof.**  Design the Lyapunov function for the entire control system as,

(64) $$\begin{array}{c} V=V_{1}+V_{\psi }+V_{u} \end{array}$$

Derivation of the above formula can be obtained,

(65) $$\begin{array}{cc} \dot{V} & ={\dot{V}}_{1}+{\dot{V}}_{\psi }+{\dot{V}}_{u}\\ & =-k_{s}x_{e}^{2}-C_{1}y_{e}^{2}-k{\tilde{g}}^{2}+\dot{g}\tilde{g}-\left(\eta_{r}-\frac{1}{2}K_{r}\right)m_{33}s_{\psi }^{2}-\left(K_{er}-\frac{1}{2}K_{r}-\frac{1}{2}\right){e^{2}}_{r}\\ & -\left(\eta_{u}-\frac{1}{2}K_{u}\right)m_{11}s_{u}^{2}-\left(K_{eu}-\frac{1}{2}K_{u}-\frac{1}{2}\right){e^{2}}_{u} \end{array}$$

According to the Young’s inequality,

(66) $$\begin{array}{c} \dot{g}\tilde{g}\leq \frac{1}{2}{\dot{g}}^{2}+\frac{1}{2}{\tilde{g}}^{2}\leq \frac{1}{2}{\bar{g}}^{2}+\frac{1}{2}{\tilde{g}}^{2} \end{array}$$

Furthermore, Formula (65) can be rewritten as,

(67) $$\begin{array}{cc} \dot{V} & \leq -k_{s}x_{e}^{2}-C_{1}y_{e}^{2}-\left(k-\frac{1}{2}\right){\tilde{g}}^{2}+\frac{1}{2}{\bar{g}}^{2}-\left(\eta_{r}-\frac{1}{2}K_{r}\right)m_{33}s_{\psi }^{2}-\left(K_{er}-\frac{1}{2}K_{r}-\frac{1}{2}\right){e^{2}}_{r}\\ & -\left(\eta_{u}-\frac{1}{2}K_{u}\right)m_{11}s_{u}^{2}-\left(K_{eu}-\frac{1}{2}K_{u}-\frac{1}{2}\right){e^{2}}_{u}\\ & \leq -2\mu V+C \end{array}$$

In the above formula, $\mu =\min\{k_{s},C_{1},\left(k-\frac{1}{2}\right),\left(\eta_{r}-\frac{1}{2}K_{r}\right)m_{33},\left(K_{er}-\frac{1}{2}K_{r}-\frac{1}{2}\right),$

$\left(\eta_{u}-\frac{1}{2}K_{u}\right)m_{11},\left(K_{\mathrm{eu}}-\frac{1}{2}K_{u}-\frac{1}{2}\right)\}$, $C=\frac{1}{2}{\bar{g}}^{2}$. Solving Equation (67), we can obtain

(68) $$\begin{array}{c} 0\leq V\leq \frac{C}{2\mu }+\left[V\left(0\right)+\frac{C}{2\mu }\right]e^{-2\mu t} \end{array}$$

Furthermore, it can be seen that V(t) is uniformly ultimately bounded closed set Ω0:=V≤CC2μ2μ. According to Formula (68), $x_{e},y_{e},\psi_{e},u_{e},r_{e}$ are uniformly ultimately bounded.

From Equations (67) and (68), we can see,

(69) $$\begin{array}{c} ∥\varepsilon ∥\leq \sqrt{\left(2V\left(0\right)-\frac{C}{\mu }\right)e^{-2\mu t}+\frac{C}{\mu }} \end{array}$$

where $\varepsilon ={\left[\begin{array}{cc} x_{e} & y_{e} \end{array}\right]}^{T}$. For any constant σε>CCμμ>0, there is a constant $T_{1}>0$, there are $∥\varepsilon ∥\leq \sigma_{\varepsilon }$ and $\forall t>T_{1}$, so that ε can reach and remain in the bounded closed set. By selecting the design parameters $k_{s}$, *k*, $\eta_{r}$, $K_{r}$, $K_{er}$, $\eta_{u}$, $K_{u}$, $K_{eu}$, the bounded closed set can be made arbitrarily small, which meets the control goal of this article. Therefore, Theorem 2 is proved. □

## 5. Simulation Obeject and Studies

In this section, the sensor applications related to the “Lanxin”, the object of study, are first introduced.The control algorithm is then compared and simulated to verify the effectiveness of the proposed Adaptive FNTSM control method based on ELOS guidance law.

### 5.1. Simulation Object

**Figure 3:**
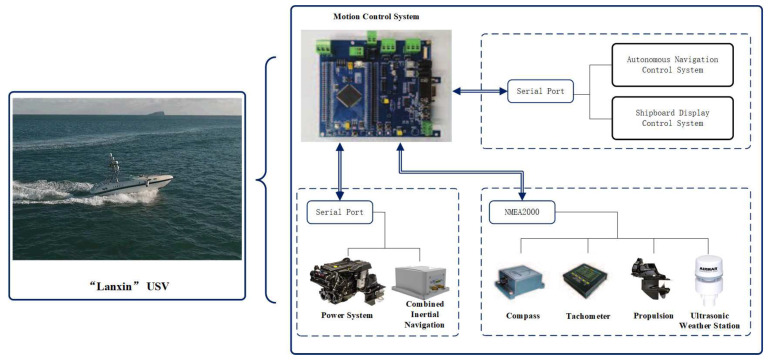
The Shipborne Sensors for “Lanxin”.

This paper uses the “Lanxin” of Dalian Maritime University as the theoretical subject of research on key technologies. As an intelligent USV that can be controlled autonomously, a variety of sensing sensors are essential. The inertial combination system can measure longitude, latitude, speed, bow angle, heading angle, longitudinal inclination angle, and other information; the steering system is equipped with angle sensors, which can accurately measure the thrust angle; through the sensor network can obtain wind speed, wind direction, engine parameters (main engine speed, fuel temperature, fuel pressure, etc.), water depth and other data. To achieve unmanned remote control of surface boats, communication devices such as DTU, radio, and 4G are also essential. The data are communicated to the control terminal via the communication devices and the controller returns the control commands to achieve the USV’s path following effect. Therefore, to achieve unmanned path following of the USV, a wealth of onboard sensors is essential. The “Lanxin” high-speed USV autonomous navigation system has the functions of navigation situational awareness, autonomous planning and decision-making, and intelligent motion control. The autonomous navigation control system is shown in Figure 3.

#### 5.1.1. Shipborne Sensors

Given the need for real-time access to information about the navigation environment and itself, navigation situational awareness is crucial. The “Lanxin” integrates a multi-sensor data acquisition and fusion onboard information processing platform to acquire the current and future status of the USV (e.g., position, bearing, speed, and acceleration) and to sense the unmanned surface boat and its surroundings based on the past and current data of the USV and the navigational status information obtained from shipborne sensors (including wind speed/direction data, etc.). The USV and its surroundings are sensed based on the past and current state of the USV as well as on information about the navigation environment (including wind speed/direction data etc.) obtained from onboard sensors. Taking into account the position, velocity, angle, and wind and wave current disturbances that are relevant for the path-following control of the USV, the GPS navigation sensors and combined inertial navigation are presented in detail.

(1) GPS Navigation Sensors

The Global Positioning System (GPS), which is a high-accuracy wireless navigation system based on artificial earth satellites, used the NEO-5Q main chip (U-blox, Zurich, Switzerland). The GPS module communicates with the microcontroller using the NMEA2000 protocol. It provides accurate position, speed, and time information anywhere in the world and near-Earth space.

(2) Combined Inertial Navigation

Combined inertial navigation used UMPOLA V18D, which integrates a variety of sensors, including triaxial gyroscopes, triaxial accelerometers, and other sensors. External auxiliary devices are also generally available. They operate simultaneously in series and can also compensate for each other’s deficiencies when using filtering algorithms. During navigation, it not only gives real-time information on the position of the USV, but also on the motion status of the USV via the attitude measurement unit, and sends the data to the USV via the serial port, accurately and quickly. Yaw angle, pitch angle, roll angle, and the corresponding angular rate can be provided and communicated via the NMEA0183 protocol.

(3) Ultrasonic Weather Station

Wind speed, a typical disturbance, is measured using the Ultrasonic Weather Station 200 WX (Airmar, Milford, NH, USA) and the disturbance data are transmitted to the controller via the CAN bus. The 200 WX weather station instrument provides accurate measurements of current weather conditions, including true wind speed and direction, air temperature and air pressure. It is also waterproof to IPX7 and has a low current consumption.

#### 5.1.2. Model Parameters

The following is to verify the effectiveness of the proposed ELOS guidance method and path-following control law. Simulation experiments are carried out with the three-degree-of-freedom under-actuated model of the “Lanxin” USV of Dalian Maritime University as the research object. The nominal physical parameters are given as follows [1], which are shown in Table 1.

Set the initial position coordinates of the USV as (0,50), the expected forward speed is 5 m/s, and the other initial states are all zero. To illustrate the superiority of the algorithm, in the guidance part, the ELOS guidance method proposed in this paper is compared with the AILOS guidance method in the literature [9]; in the control part, the fast non-singular terminal synovial membrane is compared with the ordinary non-singular terminal sliding mode control. Simulation comparisons were carried out on the models. The guidance law of AILOS is,

(70) $$\begin{array}{c} \left\{\begin{array}{c} \psi_{d}=\alpha_{k}+\tan^{-1}\left(-\frac{y_{e}}{\Delta }-\hat{\beta }\right)\\ \dot{\hat{\beta }}=\gamma \frac{U\Delta }{\sqrt{\Delta^{2}+\left(y_{e}+\Delta \hat{\beta }\right)}}y_{e} \end{array}\right. \end{array}$$

The ordinary non-singular terminal sliding mode is given as follows,

(71) $$\begin{array}{c} \left\{\begin{array}{c} s_{\psi }=\psi_{e}+\chi_{1}{\left|{\dot{\psi }}_{e}\right|}^{\frac{q_{1}}{q_{2}}}\\ s_{u}=u_{e}+\chi_{2}{\left|u_{e}\right|}^{\frac{q_{3}}{q_{4}}}+\chi_{3}\int_{}u_{e}dt \end{array}\right. \end{array}$$

Due to the obvious interaction between ship speed and sideslip angle. To verify the performance of the control algorithm designed in this paper at different sideslip angles and speeds, simulation experiments were carried out at both speeds.

### 5.2. Following a Straight Line

The expected path of design straight line follows as $S_{d}={\left[\theta ,\theta \right]}^{T}$. The design parameters are $k_{s}=10,\;\eta_{r}=2,\;K_{r}=0.0001,\;K_{er}=-500,\;k=20,\;\eta_{u}=0.1,\;K_{u}=0.0001,\;K_{eu}=-500,\;\Delta =7,\;a=\;97/99,\;\phi =\;0.01,\;L=2000\;\;,\;\alpha_{\psi }=4,\;\beta_{\psi }=1,\;\alpha_{u}=\;400,\;\beta_{u}=\;20$.

The disturbances are designed as follows,

(72) $$\begin{array}{c} \left\{\begin{array}{c} d_{u}=4000+1000\sin\left(0.8t+0.3\pi \right)+1000\cos\left(0.5t\right)\\ d_{v}=4000+500\cos\left(0.4t+0.2\pi \right)+1000\sin\left(0.4t\right)\\ d_{r}=16000+2000\sin\left(0.8t+0.2\pi \right)+500\cos\left(0.3t\right) \end{array}\right. \end{array}$$

#### 5.2.1. Moderate Speed

Controlled the USV’s speed maintained at 3 m/s.

**Figure 4:**
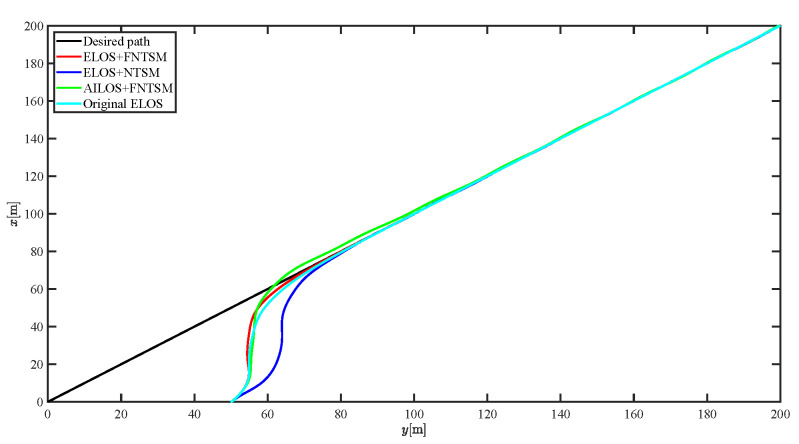
Comparison results of straight line trajectory tracking at moderate speed.

**Figure 5:**
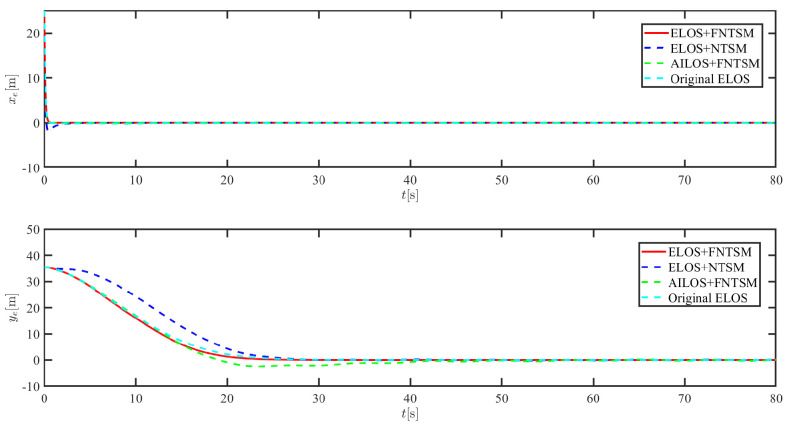
Along-track error $x_{e}$ and cross-track error $y_{e}$ at middle speed.

**Figure 6:**
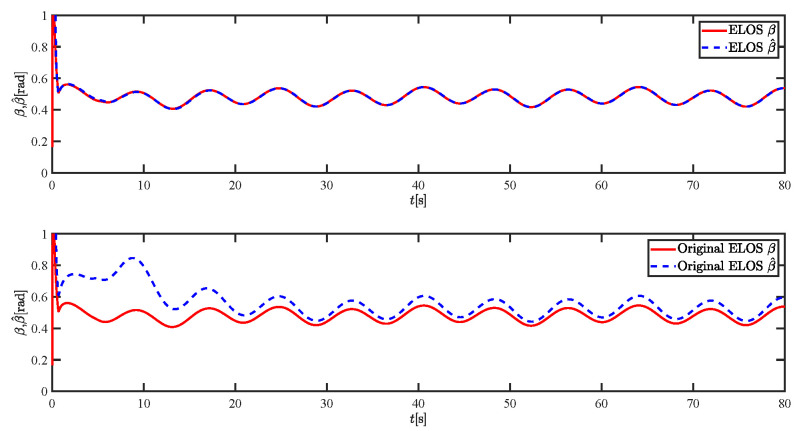
Sideslip angle estimations at moderate speed.

**Figure 7:**
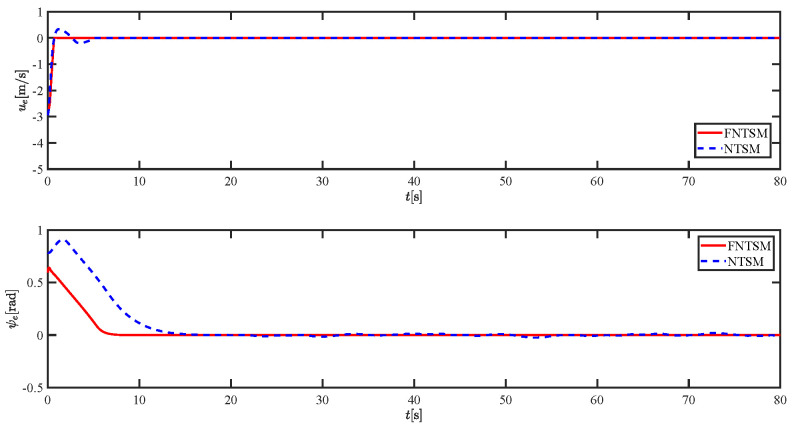
Comparison results of $u_{e}$ and $\psi_{e}$ at moderate speed.

**Figure 8:**
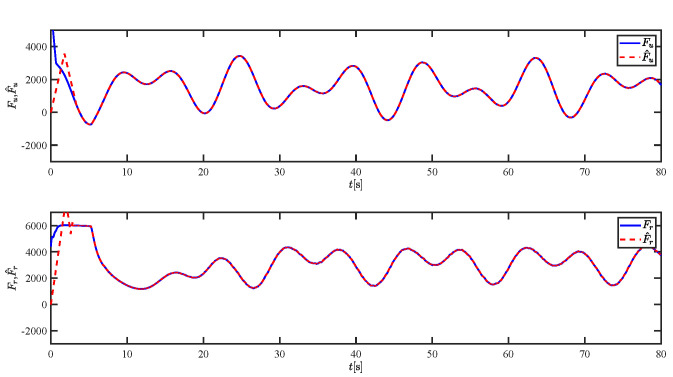
The lumped disturbances and their estimations at moderate speed.

**Figure 9:**
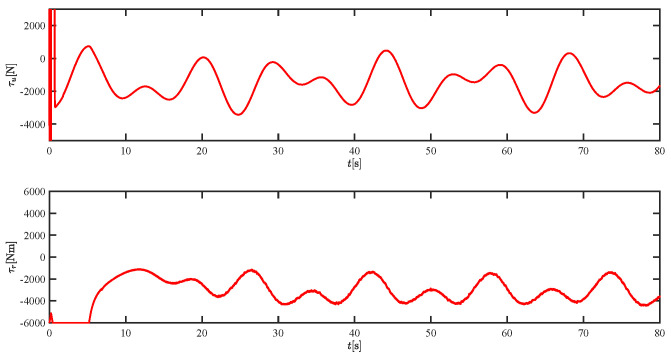
The force $\tau_{u}$ and moment $\tau_{r}$ at moderate speed.

The results of the comparison at moderate speed are given in Figure 4, Figure 5, Figure 6 and Figure 7. Figure 4 shows the difference in overall path-following effectiveness. Figure 4 and Figure 5 demonstrate that ELOS has a smaller overshoot than AILOS and that FNTSMC can track the target line path faster than NTSMC. This indicates that the combination of the ELOS guidance law and FNTSMC has a faster convergence and tracking effect. Figure 5 shows that the improved ELOS has a faster convergence rate. Due to the large lateral disturbances, it can be seen that the cross-track error convergence is more pronounced. The proposed algorithm converges to 2% accuracy in 21.68 s, while the original ELOS rate takes 24.12 s to converge to 2% accuracy with a large sideslip angle, the conventional NTSM algorithm takes 26 s to converge, and the AILOS guidance law takes 40.1 s to converge to 2% accuracy due to overshoot caused by integration. Figure 6 shows the estimation of the sideslip angle by the reduced-order ESO, which achieves an accurate estimation of the sideslip angle in a short time. Theoretically, as the gain *k* becomes larger, the observation effect will be better. However, considering the actual situation of “Lanxin”, this paper makes k = 20 in both ELOS simulations, and the algorithm proposed in this paper has a better tracking effect with a larger sideslip angle than the original ELOS with the same parameters. It is shown that the combination of the ELOS guidance method and FNTSMC has faster convergence and tracking effect. Comparing FNTSM with NTSM in the simulation environment of this paper, the convergence time of the velocity error is 3.91 s faster and the convergence time of the angular velocity error is 7.92 s faster. The control proposed in this paper can converge the velocity error to zero in a much shorter time. Meanwhile, the controller parameter ϕ is chosen as much as possible to be no less than the minimum value of 1 in order to better ensure the control effect. The most critical parameter of the adaptive term is $\varepsilon_{r}$, too large or too small will affect the accuracy and needs to be debugged based on experience. Figure 7 shows that the FNTSMC has a much faster and more responsive error convergence. The size of the parameter *L* is related to the ship model parameters, with larger model coefficients requiring an equally large *L* match. As can be seen in Figure 8, the designed finite-time lumped disturbance observer can achieve an accurate estimation of environmental disturbances and model uncertainties, improving the robustness of the control system. As shown in Figure 9, the designed auxiliary dynamic system can keep the actuated force and moment in a short-range, allowing a stable control output for the actuator even when the input is limited.

#### 5.2.2. Fast Speed

Controlled the USV’s speed maintained at 5 m/s.

**Figure 10:**
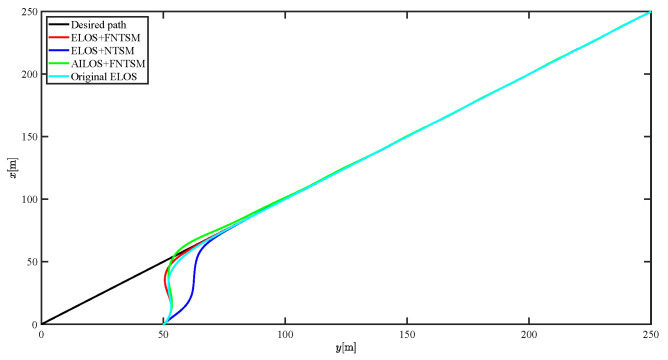
Comparison results of straight line trajectory tracking at fast speed.

**Figure 11:**
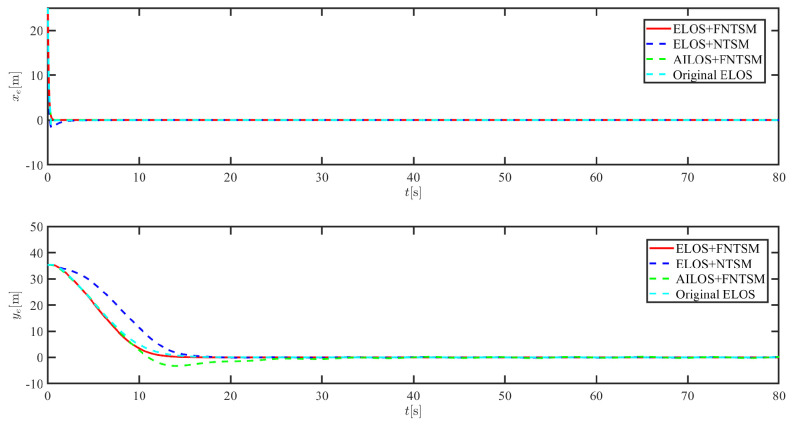
Along-track error $x_{e}$ and cross-track error $y_{e}$ at fast speed.

**Figure 12:**
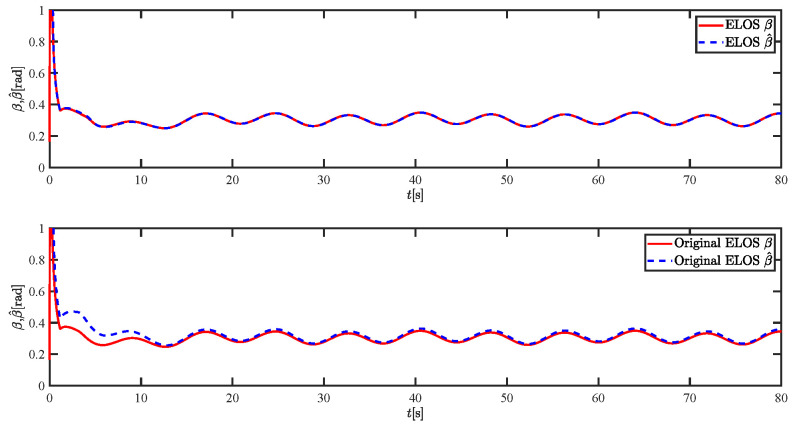
Sideslip angle estimations at fast speed.

**Figure 13:**
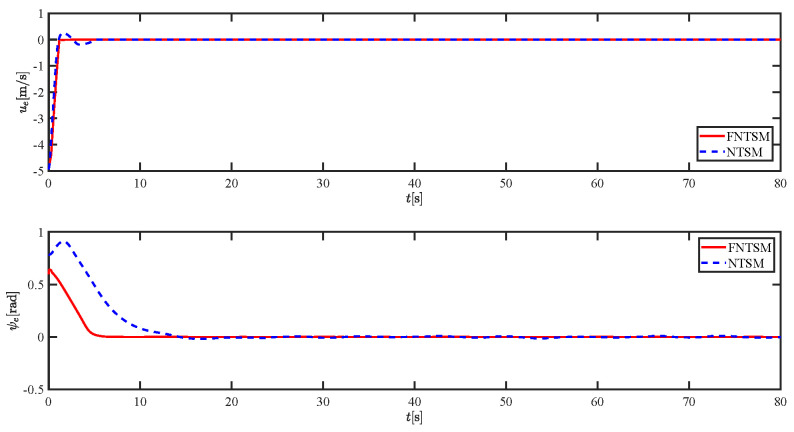
Comparison results of $u_{e}$ and $\psi_{e}$ at fast speed.

**Figure 14:**
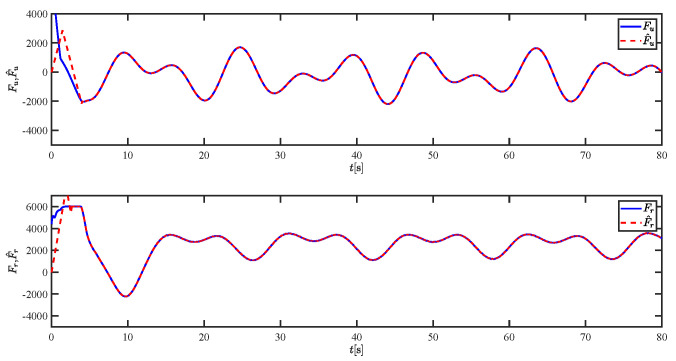
The lumped disturbances and their estimations at fast speed.

**Figure 15:**
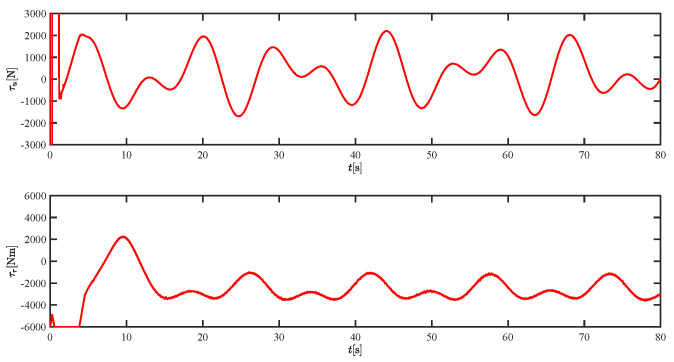
The force $\tau_{u}$ and moment $\tau_{r}$ at fast speed.

Simulation results at fast speed are given in Figure 10, Figure 11, Figure 12, Figure 13, Figure 14 and Figure 15. Stable tracking of the linear path is still achieved with unchanged parameters. The designed reduced-order ESO and finite-time lumped disturbance observer provide an accurate estimation of the sideslip angle concerning the total set disturbance. This demonstrates the strong robustness of the system. To quantify the differences, the IAE function is selected below as a performance indicator to evaluate the control strategy. IAE represents the absolute value of the error as an integral over time, where $IAE=\int_{0}^{+\infty }\left|e(t)\right|dt$. A smaller value represents the system with a smaller cumulative error.

As shown in Table 2, the algorithm proposed in this paper has significant performance advantages considering both $x_{e}$ and $y_{e}$. With better control performance.

### 5.3. Following a Curve Line

The expected path of design straight line follows as $S_{d}={\left[30\sin(\frac{\theta }{30})+\theta ,\theta \right]}^{T}$. The design parameters are $k_{s}=10,\;\eta_{r}=2,\;K_{r}=0.0001,\;K_{er}=-500,\;k=20,\;\eta_{u}=0.1,\;K_{u}\;=\;0.0001,\;$$K_{eu}=-500,\;\Delta =7,\;a=\;97/99,\;\phi =\;0.01,L=2000\;\;,\alpha_{\psi }=4,\;\beta_{\psi }=1,\;\alpha_{u}=\;400,\;\beta_{u}=\;20$.

#### 5.3.1. Moderate Speed

Controlled the USV’s speed maintained at 3 m/s.

**Figure 16:**
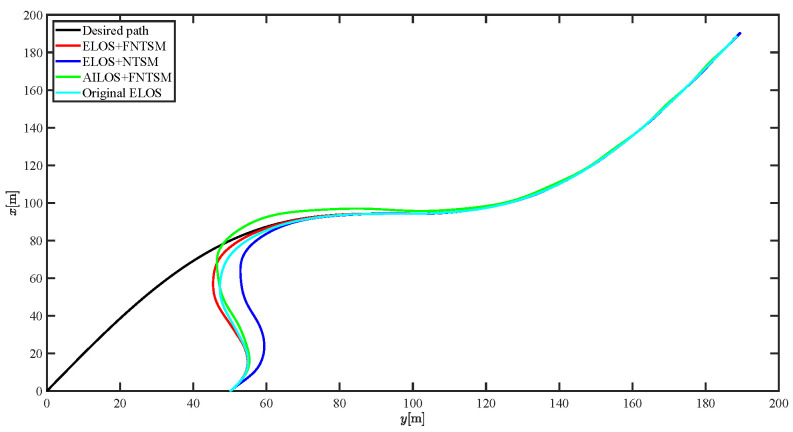
Comparison results of curve line trajectory tracking at moderate speed.

**Figure 17:**
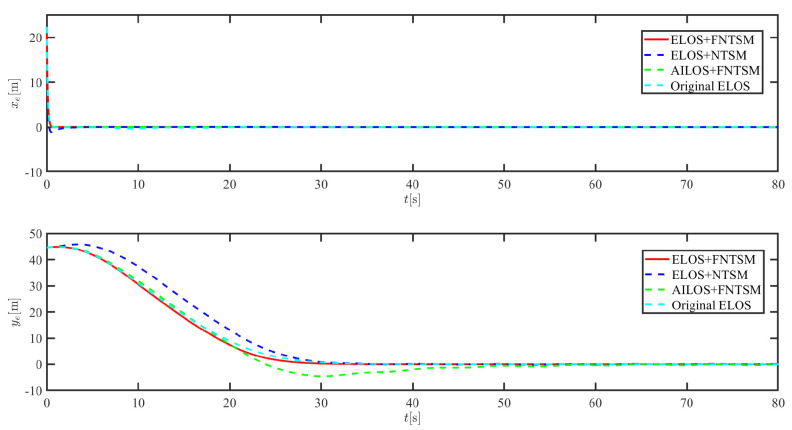
Along-track error $x_{e}$ and cross-track error $y_{e}$ at moderate speed.

**Figure 18:**
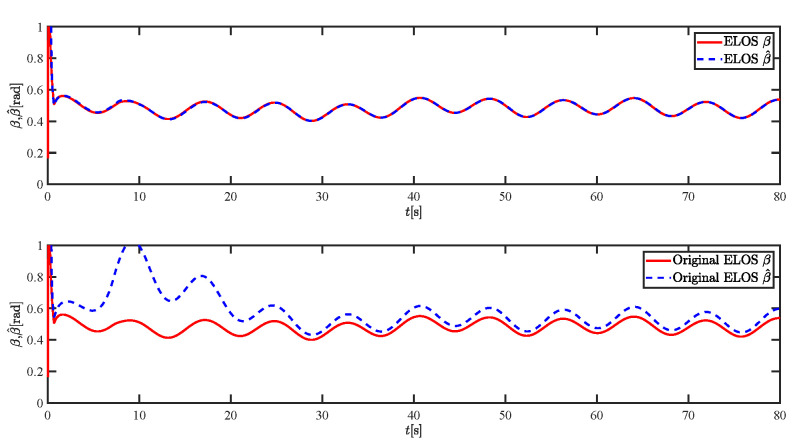
Sideslip angle estimations at moderate speed.

**Figure 19:**
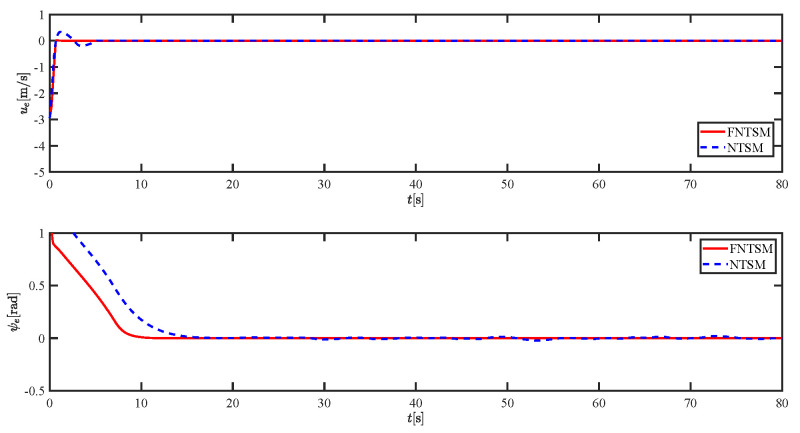
Comparison results of $u_{e}$ and $\psi_{e}$ at moderate speed.

**Figure 20:**
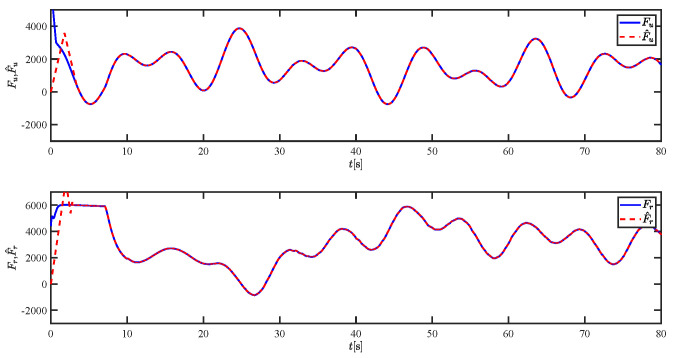
The lumped disturbances and their estimations at moderate speed.

**Figure 21:**
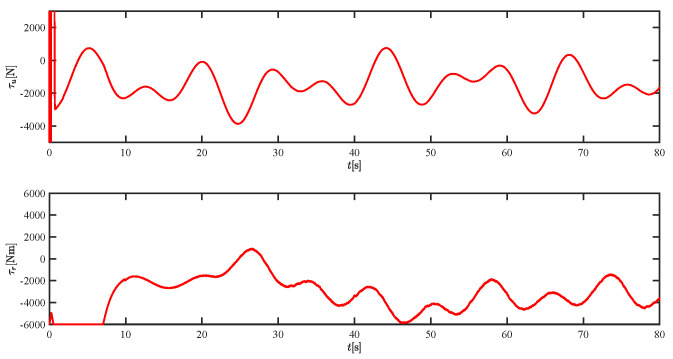
The force $\tau_{u}$ and moment $\tau_{r}$ at moderate speed.

The results of the comparison at moderate speed are given in Figure 16, Figure 17, Figure 18 and Figure 19. As the design of the paths becomes complex, the combined control of ELOS and FNTSM has a more significant advantage in terms of convergence speed and has smaller overshoot and tracking errors. The estimates shown in Figure 18 and Figure 20 accurately track the sideslip angle and lumped disturbances. As can be seen in Figure 18, the original ELOS has a significant steady-state error for this degree of sideslip angle. The adjustment of parameter *k* improves the speed of convergence of the drift angle estimate, but there is no way to compensate for the error caused by the small-angle approximation. The graph of the actuator is given in Figure 21.

#### 5.3.2. Fast Speed

Controlled the USV’s speed maintained at 5 m/s.

**Figure 22:**
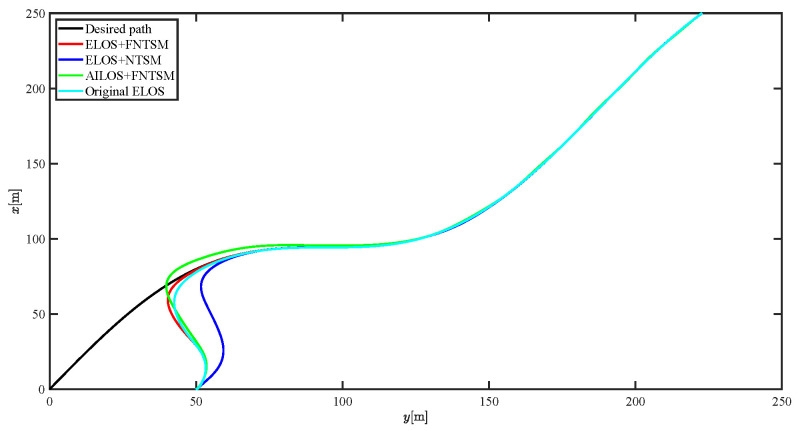
Comparison results of curve line trajectory tracking at fast speed.

**Figure 23:**
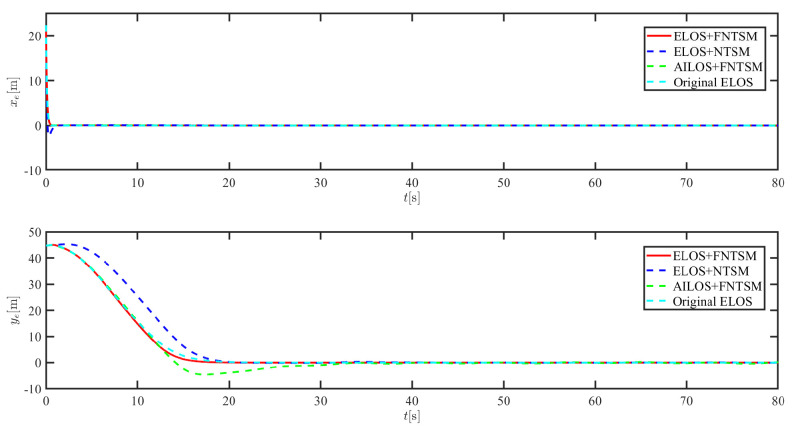
Along-track error $x_{e}$ and cross-track error $y_{e}$ at fast speed.

**Figure 24:**
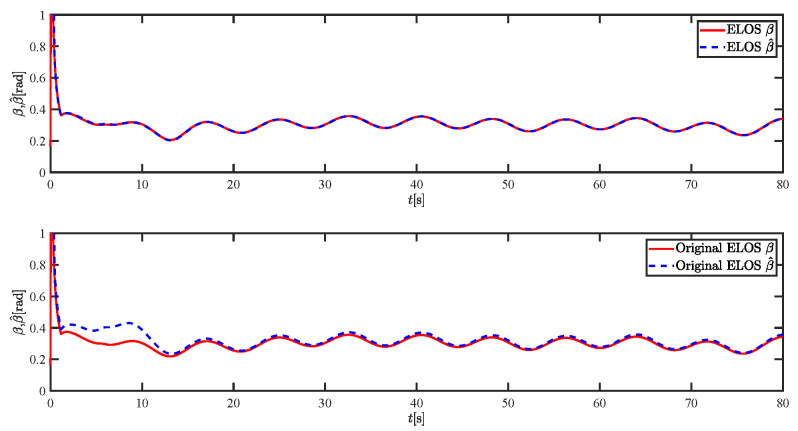
Sideslip angle estimations at fast speed.

**Figure 25:**
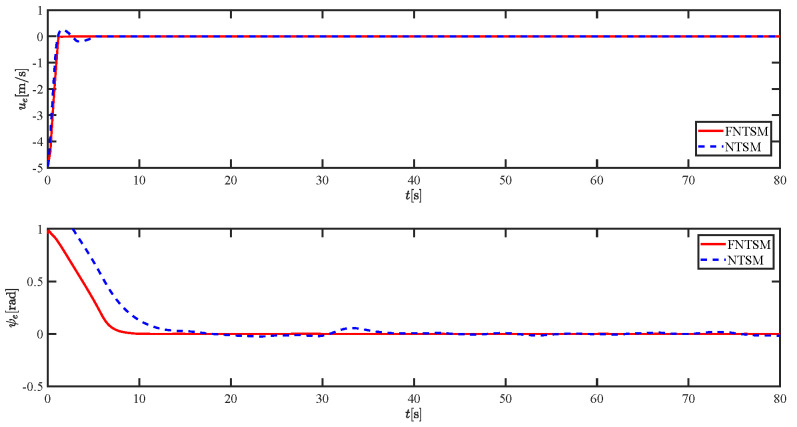
Comparison results of $u_{e}$ and $\psi_{e}$ at fast speed.

**Figure 26:**
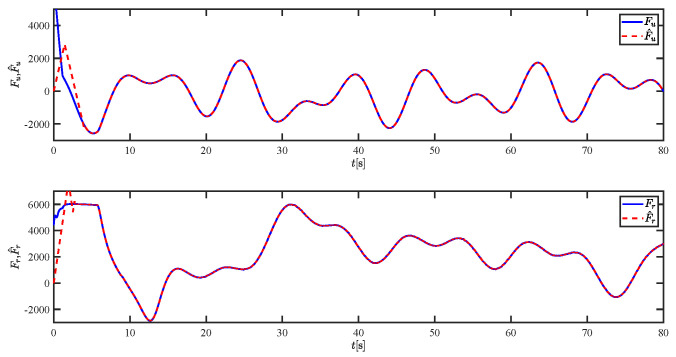
The lumped disturbances and their estimations at fast speed.

**Figure 27:**
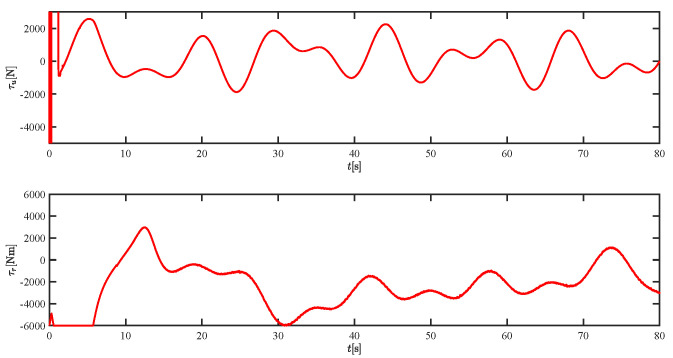
The force $\tau_{u}$ and moment $\tau_{r}$ at fast speed.

Simulation results at fast speed are given by Figure 22, Figure 23, Figure 24, Figure 25, Figure 26 and Figure 27. There are fluctuations as the USV reaches the curve inflection point. Figure 25 shows that the designed FNTSM controller can control the USV stabilization speed error at a faster rate. As shown in Figure 24, the sideslip angle is kept between 0.2 and 0.35. In this range, the algorithm proposed in this paper has a much better fit. According to the IAE function in Table 3, the algorithm proposed in this paper still has a clear advantage. Figure 27 shows that the designed auxiliary dynamic system can guarantee fast and stable control input even when there are control quantities above the threshold. In summary, the ELOS-based adaptive path-following control algorithm presented in this paper is efficient for the path-following problem of uncertain USVs under unknown time-varying disturbances and time-varying large sideslip angles.

### 5.4. Severe Disturbance

The quality of the sea state is related to the frequency and amplitude of the waves. In general, the worse the sea conditions, the lower the frequency and the higher the amplitude of the waves. This section presents a simulation study of severe disturbance. The disturbance is given as follows,

(73) $$\begin{array}{c} \left\{\begin{array}{c} d_{u}=4000+2000\sin\left(0.4t+0.15\pi \right)+2000\cos\left(0.15t\right)\\ d_{v}=4000+1000\cos\left(0.2t+0.1\pi \right)+2000\sin\left(0.2t\right)\\ d_{r}=16000+4000\sin\left(0.4t+0.15\pi \right)+1000\cos\left(0.15t\right) \end{array}\right. \end{array}$$

**Figure 28:**
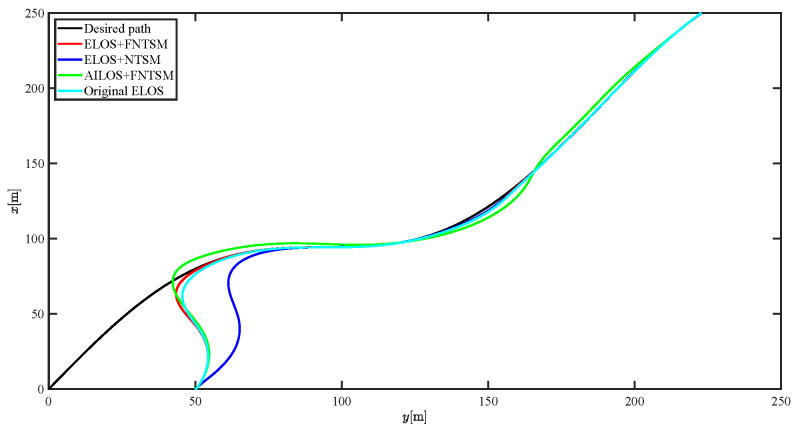
Comparison results of curve line trajectory tracking under severe disturbance.

**Figure 29:**
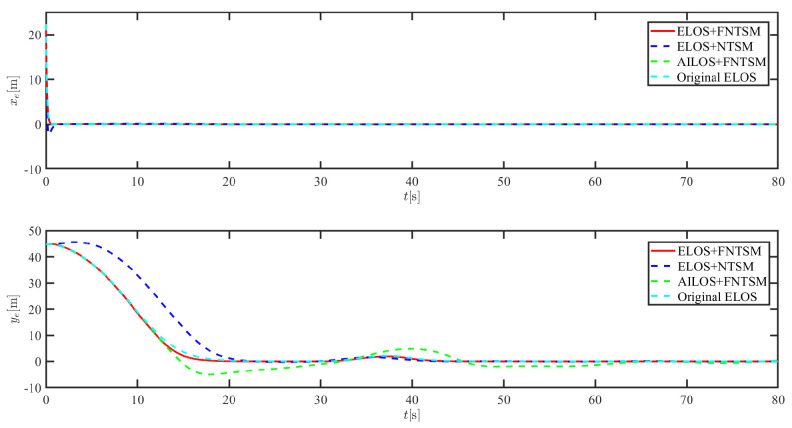
Along-track error $x_{e}$ and cross-track error $y_{e}$ under severe disturbance.

**Figure 30:**
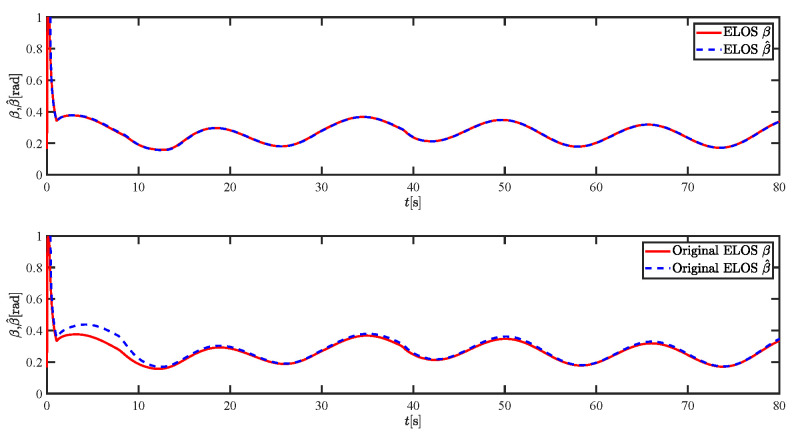
Sideslip angle estimations under severe disturbance.

**Figure 31:**
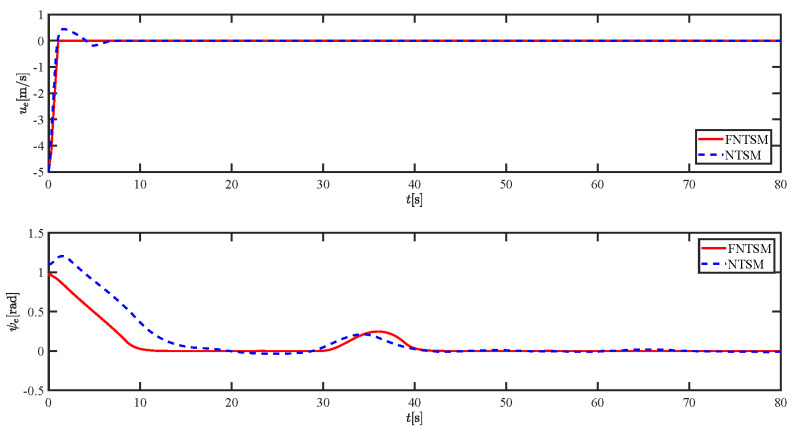
Comparison results of $u_{e}$ and $\psi_{e}$ under severe disturbance.

**Figure 32:**
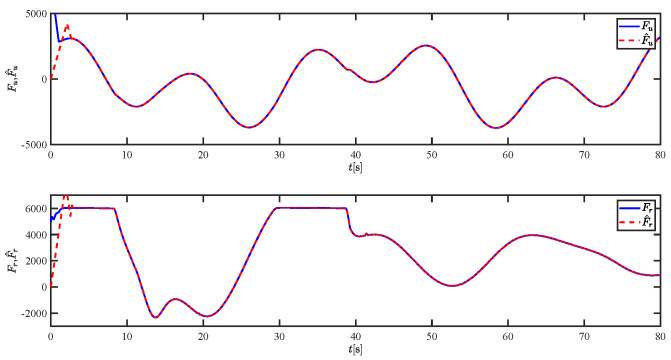
The lumped disturbances and their estimations under severe disturbance.

**Figure 33:**
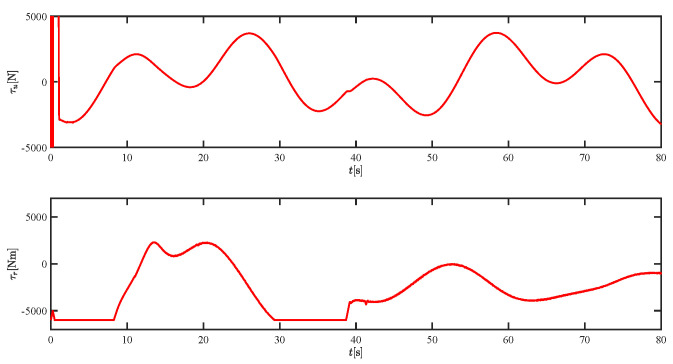
The force $\tau_{u}$ and moment $\tau_{r}$ under severe disturbance.

Figure 28 shows that the control algorithm proposed in this paper still performs well under severe disturbances. In particular, there is no significant overshoot at the inflection points of the curve path. Figure 29 shows the convergence speed of $x_{e}$ and $y_{e}$. Combined with Table 4, it can be seen from Figure 28, Figure 29, Figure 30, Figure 31, Figure 32 and Figure 33, that the improved ELOS in this paper has a strong robustness.

## 6. Conclusions

In this paper, an adaptive path-following control strategy based on ELOS is proposed. In the guidance section, a reduced-order ESO is introduced to estimate the time-varying sideslip angle and to avoid the errors arising from the small-angle approximation, thus obtaining the desired heading angle. In the dynamics controller section, a finite-time disturbance observer-based FNTSM controller is designed. In this, an auxiliary dynamic system is introduced to consider actuator saturation, thus enhancing the practicality of the system in real situations. In addition, the introduction of an adaptive term enhances the robustness of the system. The improved ELOS does not rely on the small-angle approximation principle and thus extends the range of application and accuracy. The proposed adaptive FNTSM control algorithm is the first to be introduced for Underactuated USV path-following control, allowing for faster convergence of tracking errors and weakening of controller chattering. Simulation experiments demonstrate that the proposed control strategy, with the selection of appropriate design parameters, can make the path-following closed-loop control system guaranteed uniform ultimate boundedness for all signals.

Future work includes the following three areas. One is that interference observers with faster convergence can be investigated. The second is that the path-following control of a single ship can be extended to a multi-ship formation algorithm. The third, the model in this paper does not take into account ocean currents. Increasing the complexity of the model is also one of the directions of development.

## Figures and Tables

**Table 1:** The “LanXin” USV Parameters.

Parameters	Value
Length Between Perpendiculars	7.02 m
Breadth	2.60 m
Speed	≤35 kn
Draft (full load)	0.32 m
Block Coefficient	0.6976
Displacement (full load)	2.73 m${}^{3}$
Rudder Area	0.2091 m${}^{2}$
Distance Between Barycenter and Center	0.35 m

**Table 2:** Performance indicator of path-following (straight).

Performance Indicator	ELOS + FNTSM	ELOS + NTSM	AILOS + FNTSM	Original ELOS
IAE($x_{e}$)	3.5355	4.8827	3.9374	6.0828
IAE($y_{e}$)	210.0264	293.8310	243.2823	220.8267

**Table 3:** Performance indicator of path-following (curve).

Performance Indicator	ELOS + FNTSM	ELOS + NTSM	AILOS + FNTSM	Original ELOS
IAE($x_{e}$)	2.6482	3.1787	3.0558	4.3136
IAE($y_{e}$)	280.4689	487.6118	423.8859	383.7557

**Table 4:** Performance indicator of path-following (curve).

Performance Indicator	ELOS + FNTSM	ELOS + NTSM	AILOS + FNTSM	Original ELOS
IAE($x_{e}$)	2.8795	3.9217	3.3716	3.7925
IAE($y_{e}$)	415.6106	584.2268	518.8231	429.1937

## Data Availability

Not applicable.

## Abbreviations

The following abbreviations are used in this manuscript:

USV	unmanned surface vehicle
LOS	line-of-sight
SMC	sliding mode control
FNTSMC	fast non-singular terminal sliding mode control
SGPFS	semi-global practical finite-time stability
GPS	global positionting system
DTU	data terminal unit
DOF	degree of freedom
NTSMC	Fast non-singular terminal sliding mode control
